# Halofuginone Disrupted Collagen Deposition via mTOR‐eIF2α‐ATF4 Axis to Enhance Chemosensitivity in Ovarian Cancer

**DOI:** 10.1002/advs.202416523

**Published:** 2025-03-24

**Authors:** Wenxin Li, Yenan Wu, Yanan Zhang, Wenyan Gao, Xin Li, Haixia Luo, Mengmeng Lu, Zhihua Liu, Aiping Luo

**Affiliations:** ^1^ State Key Lab of Molecular Oncology National Cancer Center/National Clinical Research Center for Cancer/Cancer Hospital Chinese Academy of Medical Sciences and Peking Union Medical College 17 Nanli Panjiayuan, Chaoyang District Beijing 100021 China; ^2^ Department of Obstetrics and Gynecology Peking University Third Hospital 38 Xueyuan Rd, Haidian District Beijing 100191 China; ^3^ Department of Gynecological Oncology National Cancer Center/National Clinical Research Center for Cancer/Cancer Hospital Chinese Academy of Medical Sciences and Peking Union Medical College 17 Nanli Panjiayuan, Chaoyang District Beijing 100021 China

**Keywords:** cancer‐associated fibroblasts (CAFs), COL1A1, extracellular matrix, fibrosis, halofuginone, ovarian cancer

## Abstract

The interplay between cancer‐associated fibroblasts (CAFs) and extracellular matrix (ECM) mediates progress, metastasis, and therapy resistance. However, strategy of targeting ECM remodeling to enhance chemosensitivity in ovarian cancer remains elusive. Here, a 22‐gene matrisome signature predicts chemotherapy response and survival in ovarian cancer. The dense, collagen‐rich ECM secreted by CAFs harbors more M2 tumor‐associated macrophages (TAMs) than the looser ECM based on single cell RNA‐seq (scRNA‐seq) of ovarian cancer, suggesting the promising approach of targeting collagen to remodel ECM. An integrated analysis identifies collagen type I alpha 1 chain (COL1A1) as a major component of the ECM that contributes to chemoresistance and poor prognosis, highlighting its potential as a therapeutic target. Halofuginone (HF), a clinically active derivative of febrifugine, is identified as a COL1A1‐targeting natural compound by screening the Encyclopedia of Traditional Chinese Medicine (ETCM). Mechanistically, HF inhibits COL1A1 production via the mTOR‐eIF2α‐ATF4 axis in CAFs. Notably, HF disrupts collagen deposition and promotes CD8+ T cell infiltration, partially via M2‐M1 macrophage polarization to enhance chemosensitivity. Overall, the findings suggest that HF combined with chemotherapy is a promising and effective treatment for ovarian cancer.

## Graphical abstract

COL1A1, a major component of ECM, contributes to chemoresistance and poor prognosis in ovarian cancer. Halofuginone (HF) attenuates CAF‐secreted COL1A1 through mTOR‐eIF2α‐ATF4 axis. Notably, HF disrupts collagen deposition to form the looser ECM and facilitates CD8+ T cell infiltration, partially via M2‐M1 macrophage polarization to enhance chemosensitivity. Overall, targeting ECM offers a promising therapeutic approach for ovarian cancer.

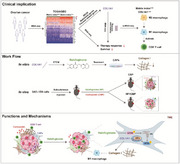

## 1 Introduction

Ovarian cancer is a highly aggressive disease, ranking third in annual incidence among female reproductive system tumors.^[^ ^1^, ^2^ ^]^ Due to the lack of corresponding symptoms in the early stages, ≈70% of ovarian cancer patients are diagnosed at an advanced stage.^[^ ^3^, ^4^ ^]^ Platinum‐based chemotherapy is the standard of care for ovarian cancers, but treatment resistance often develops.^[^ ^5^ ^]^ Two major clinical problems that ovarian cancer faces today are metastasis and chemoresistance.^[^ ^2^ ^]^ Chemoresistance is mainly related to tumor heterogeneity, alteration of tumor microenvironment (TME), activation of drug efflux pumps, and detoxification enzymes caused by genetic mutations.^[^ ^6^ ^]^ Therefore, overcoming chemoresistance could improve therapeutic efficacy of ovarian cancer.

TME is composed of cellular and non‐cellular components.^[^ ^7^, ^8^ ^]^ Stromal cells, particularly cancer‐associated fibroblasts (CAFs) and tumor‐associated macrophages (TAMs), secrete cytokines and chemokines to induce metabolic reprogramming and extracellular matrix (ECM) remodeling through synthesis, degradation, reassembly, and chemical modification.^[^ ^9^ ^]^ ECM, defined as a “matrisome”, includes fibrillar proteins (collagens, glycoproteins, and proteoglycans), ECM‐affiliated proteins, ECM regulators, and secreted factors that cooperate to assemble and remodel extracellular matrix and bind to cells through ECM receptors.^[^ ^10^, ^11^, ^12^, ^13^, ^14^ ^]^ Transcriptomic and proteomic analysis revealed the composition and complexity of matrisome, as well as the dynamic nature of ECM remodeling that drives ovarian cancer metastasis and chemoresistance.^[^ ^15^, ^16^ ^]^ 22 matrisome genes distinguished patients with a shorter overall survival in ovarian and 12 other primary solid cancers.^[^ ^15^ ^]^ Based on these findings, we postulated that ECM is associated with chemoresistance in ovarian cancer.

CAFs function as myofibroblasts and are activated by a complex series of signals that lead to the excessive ECM deposition and ECM remodeling, resulting in fibrosis or desmoplasia, diminished antitumor immune infiltration, and even chemoresistance.^[^ ^17^, ^18^, ^19^ ^]^ Fibrosis is not classified as a disease but rather as a pathological process that commonly arises from chronic inflammation. The excessive ECM deposition is a hallmark of fibrosis and cancer.^[^ ^20^ ^]^ The dense fibrotic matrix serves to create a physical barrier, providing a unique environment that impedes drug accumulation and immune cell infiltration, resulting in an “immunologically cold” TME.^[^ ^21^, ^22^ ^]^ Ovarian cancer patients with a high proportion of ECM tend to tolerate platinum chemotherapy.^[^ ^23^ ^]^ Recent studies showed that TAMs respond to the stiffened fibrotic TME by initiating a collagen biosynthesis program directed by transforming growth factor‐β (TGFβ),^[^ ^24^ ^]^ and targeting the HSP47‐collagen axis inhibits brain metastasis by reversing M2 microglial polarization and restoring anti‐tumor immunity.^[^ ^25^ ^]^ These findings indicated that targeting collagen could eliminate fibrosis to facilitate immune cell infiltration.

ECM components, such as collagen, produced by CAFs contribute to chemoresistance and promote immunosuppression by increasing tissue stiffness. Targeting ECM sensitizes chemotherapy in ovarian cancer remains unelusive. Here, we demonstrated a 22‐gene matrisome signature (termed Matrix index) for predicting prognosis and chemoresistance in ovarian cancer. Among them, collagen type I alpha 1 chain (COL1A1), a major component of ECM, was significantly upregulated in Matrix index^High^ group and resist chemotherapy, indicating that COL1A1 acts as a potential therapeutic target in ovarian cancer. A major structural component of tumor ECM is fibrillar collagens. Notably, high levels of Matrix index or COL1A1 expression is positively associated with the number of M2 macrophages in clinical ovarian cancer tissues. Therefore, we further screened for potential natural compounds targeting COL1A1 and identified halofuginone (HF), a clinically active derivative of febrifugine using Encyclopedia of Traditional Chinese Medicine (ETCM).^[^ ^25^ ^]^ HF is a specific inhibitor of collagen type I (Col1) synthesis and attenuates osteoarthritis (OA) by inhibition of TGFβ activity.^[^ ^26^, ^27^ ^]^ Therefore, we selected HF as a compound of targeting COL1A1. Mechanistically, HF inhibits CAF‐secreted COL1A1 through mTOR‐eIF2α‐ATF4 axis, enhances chemosensitivity by disrupting collagen deposition, and facilitates CD8+ T cell infiltration in vivo. Our findings offer new insights into treatment of ovarian cancer.

## 2 Result

### 2.1 A 22‐Gene Matrisome Signature for Predicting Chemotherapy Response and Prognosis

**Figure 1:**
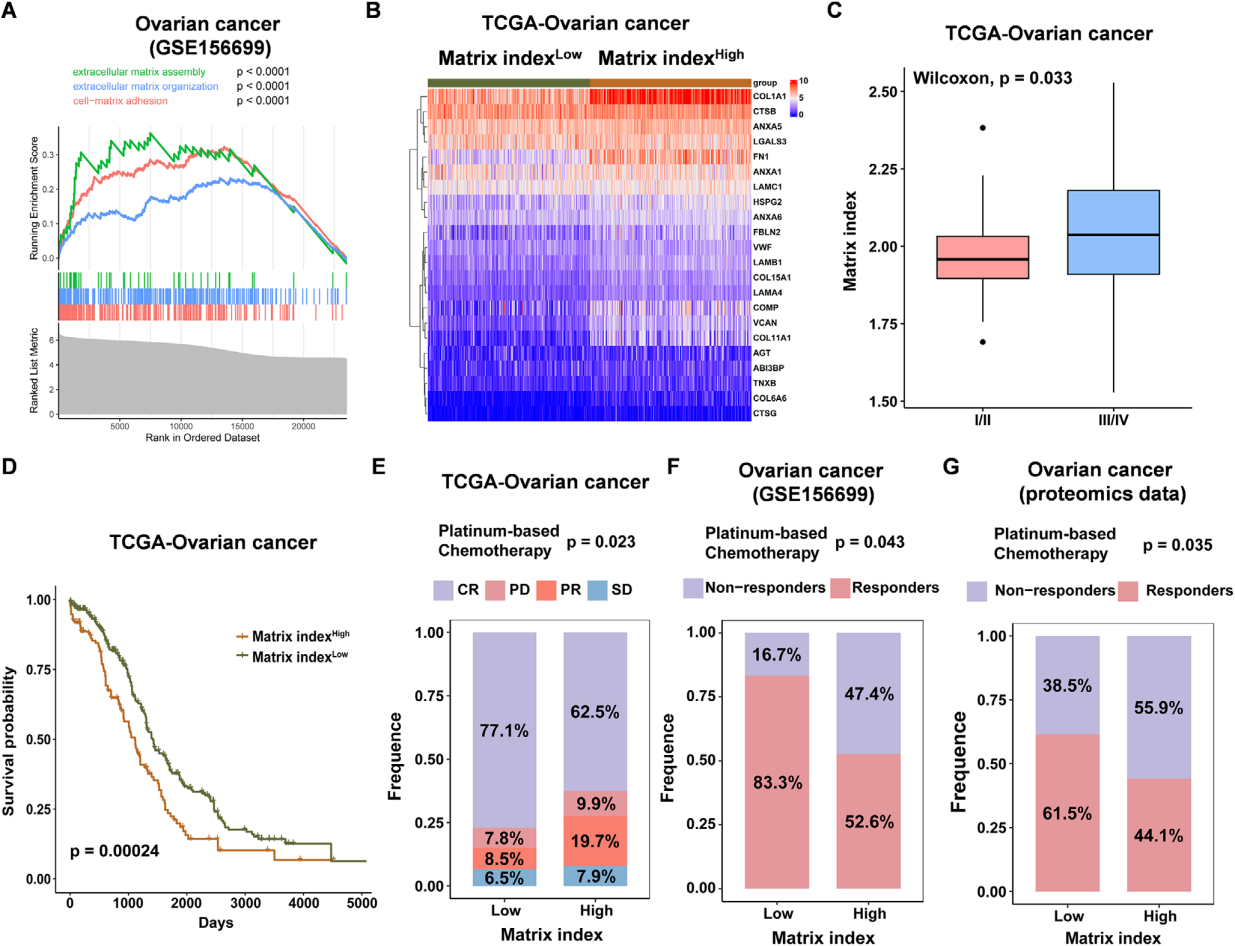
A 22‐gene matrisome signature predicts chemotherapy response and prognosis in ovarian cancer. A) GSEA analysis of differential ECM‐associated pathways in resistant and sensitive groups using GSE156699 dataset. B) Patients were divided into Matrix index^High^ group and Matrix index^Low^ group based on a 22‐gene matrisome signature using TCGA ovarian cancer dataset. C) Analysis of correlation between clinical stage and Matrix index in ovarian cancer from TCGA dataset. D) Correlation between Matrix index and overall survival of patients with ovarian cancer from TCGA dataset. E–G) Analysis of correlation between chemotherapy response and Matrix index in ovarian cancer from E) TCGA, F) GSE156699, and G) proteomics datasets.

Biochemical and physical ECM signals affect tumor formation, invasion, metastasis, and therapy resistance. Since 2012, emerging proteomic profiling has revealed the features and heterogeneity of ECM in primary tumors, distinct metastatic sites, before and after chemotherapy.^[^ ^16^, ^28^ ^]^ To explore the key pathways involved in chemoresistance, we first analyzed differentially expressed genes (DEGs) between chemo‐resistant and sensitive groups using the Gene Expression Omnibus dataset (GEO, GSE156699). Gene Ontology (GO) analysis revealed that a subset of DEGs were significantly enriched in ECM‐associated pathways, including “cell‐Matrix adhesion, ECM assembly, organization, and constituent” (**Figure** **1**A), indicating that chemoresistance was associated with the abnormal ECM. The previous study indicated that 22 matrisome genes discriminated patients with a shorter overall survival in ovarian cancer and 12 other primary solid cancers.^[^ ^15^ ^]^ To explore whether ECM was involved in regulating chemotherapy response in ovarian cancer, we first divided these patients into Matrix index^High^ group and Matrix index^Low^ group based on expression of 22 matrisome genes in the TCGA ovarian cancer dataset and GSE156699 (Figure 1B; Figure S1, Supporting Information). As shown in Figure 1C, patients with the clinical T3‐4 stage exhibited a high Matrix index (p = 0.033). Patients with high Matrix index had significantly shorter survival than patients with low Matrix index (p = 0.00024, Figure 1D). These results suggest that Matrix index may be a valuable tool for evaluating the clinical characteristics and prognosis in ovarian cancer.

Chemotherapy itself enhances resistance by progressively changing the cancer cell‐intrinsic adhesion signaling and cell‐surrounding ECM. Emerging studies have shown that the evolution of cancer cell and ECM crosstalk critically affects metastasis and therapeutic resistance.^[^ ^14^, ^16^, ^29^, ^30^ ^]^ Therefore, we performed the correlation analysis between a Matrix index and chemotherapy response in ovarian cancer from TCGA dataset, GSE156699, and ovarian cancer proteomics data^[^ ^31^ ^]^. Our finding showed that those patients with high Matrix index exhibited chemoresistance. However, in ovarian cancer patients exhibiting a low Matrix index, a relatively high response rate to chemotherapy was observed, with percentages of 77.1%, 83.3%, and 61.5%, respectively (Figure 1E–G). These results suggest that a 22‐gene matrisome signature could predict response to chemotherapy in ovarian cancer.

### 2.2 COL1A1 Confers Carboplatin Resistance

**Figure 2:**
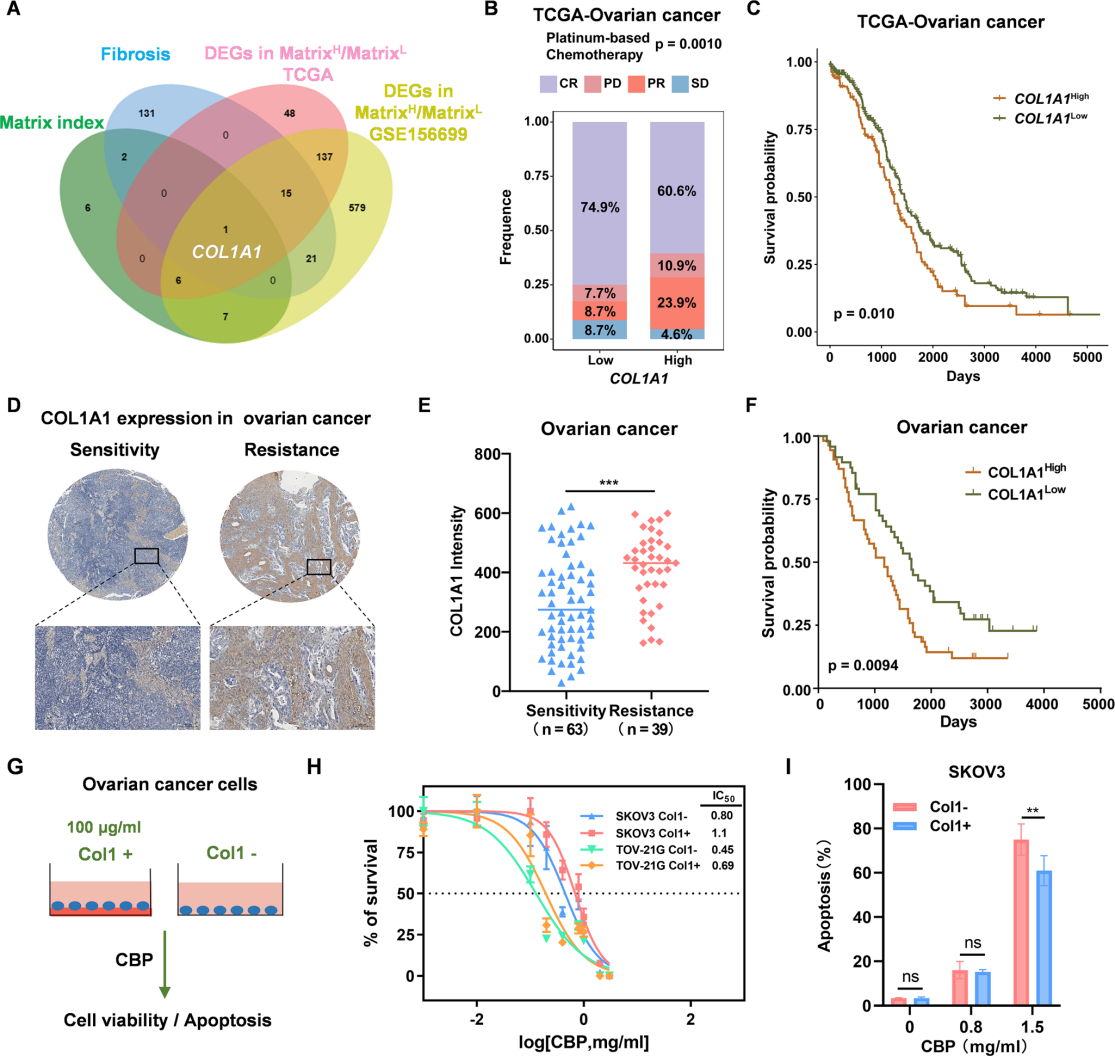
COL1A1 is associated with chemoresistance and poor prognosis in ovarian cancer. A) Venn diagram showing the overlap of Matrix index (22 matrisome genes), fibrosis‐related genes (n = 167), DEGs in the Matrix index^High^/Matrix index^Low^ group in ovarian cancer from TCGA and GSE156699 datasets. B) Correlation between COL1A1 expression and chemotherapy response in ovarian cancer from TCGA dataset. C) Correlation between COL1A1 expression and survival in ovarian cancer from TCGA dataset. D–F) COL1A1 expression in ovarian cancer was detected by immunohistochemistry (IHC) using tissue microarrays. D) Representative IHC images for COL1A1 expression in carboplatin (CBP) sensitive and resistant groups (Scale bar, 100 µm); E) Analysis of COL1A1 expression in CBP sensitive and resistant groups in ovarian cancer; F) Analysis of correlation between COL1A1 expression and survival in ovarian cancer. G) Schematic of cell viability assay treated with CBP, with or without Col1. H) SKOV3 and TOV‐21G cells were seeded in 96‐well plates coated with Col1 (100 µg mL⁻^1^) or without, and were treated with CBP after 24 h. Cell viability was determined by CCK8 assay. I) SKOV3 cells were seeded in 6‐well plates coated with Col1 (100 µg mL⁻^1^) or without, and treated with different concentration of CBP as indicated. Apoptosis was detected by flow cytometry 24 h after treatment. Data was presented as mean ± SD (n = 3). Statistical analysis was performed by two‐tailed, unpaired Student's t‐test and two‐way ANOVA with Tukey's test, **p < 0.01, ***p < 0.001.

Given that ECM is associated with prognosis and chemoresistance in ovarian cancer. Therefore, it is urgent to identify the key genes that mediate ECM remodeling. CAFs function as myofibroblasts and facilitate the excessive deposition ECM proteins and ECM remodeling,^[^ ^18^, ^19^ ^]^ while the deposition ECM could lead to fibrosis.^[^ ^32^ ^]^ A series of fibrosis‐related genes was used to evaluate correlation between fibrosis and therapy response in human cancer (http://herb.ac.cn/). Therefore, to identify ECM‐associated genes, we first analyzed DEGs between Matrix index^High^ group and Matrix index^Low^ group using GSE156699 and TCGA datasets (Table S1, Supporting Information). Subsequently, we performed an integrative analysis based on 22 matrisome genes, fibrosis‐related genes (n = 167), and DEGs in Matrix^H^/Matrix^L^ in TCGA and GSE156699 datasets, and identified COL1A1 (**Figure** **2**A).

Collagen, the major component of ECM, mediates the crosstalk of cancer cells and ECM.^[^ ^33^ ^]^ Deposition of fibrillar collagen leads to stiffening of tumor tissue and promotes chemoresistance. To evaluate the critical role of COL1A1 in ovarian cancer, we performed the following analysis using TCGA ovarian cancer dataset, GSE156699, and Kaplan‐Meier Plotter. There was a relatively high complete response (CR) rates of 74.9% after chemotherapy in ovarian cancer patients with a low Matrix index (Figure 2B). Notably, high COL1A1 expression was associated with poor survival in ovarian cancer (Figure 2C; Figure S2A,B, Supporting Information). In addition, we further examined COL1A1 expression in ovarian cancer tissue microarray by IHC. As shown in Figure 2D, COL1A1 protein was mainly localized in ECM. Consistent with the above result, high COL1A1 protein expression was also correlated with chemoresistance and poor prognosis in ovarian cancer (Figure 2E,F). Therefore, we selected COL1A1 for further investigation.

To further evaluate whether COL1A1 mediates chemoresistance, we pretreated 96‐well plates with 100 µg mL⁻^1^ Col1, and then seeded ovarian cancer cells SKOV3 and TOV‐21G respectively, and ultimately evaluated the IC50 value (Figure 2G). As shown in Figure 2H, collagen exhibited resistance to the cytotoxicity of CBP using CCK8 assay. Furthermore, we observed that collagen inhibited CBP‐induced apoptosis in ovarian cancer cells SKOV3 and TOV‐21G (Figure 2I; Figure S3, Supporting Information). Collectively, these findings indicate that COL1A1 mediates chemoresistance and prognosis in ovarian cancer.

### 2.3 Halofuginone Disrupts CAFs‐Secreted COL1A1

**Figure 3:**
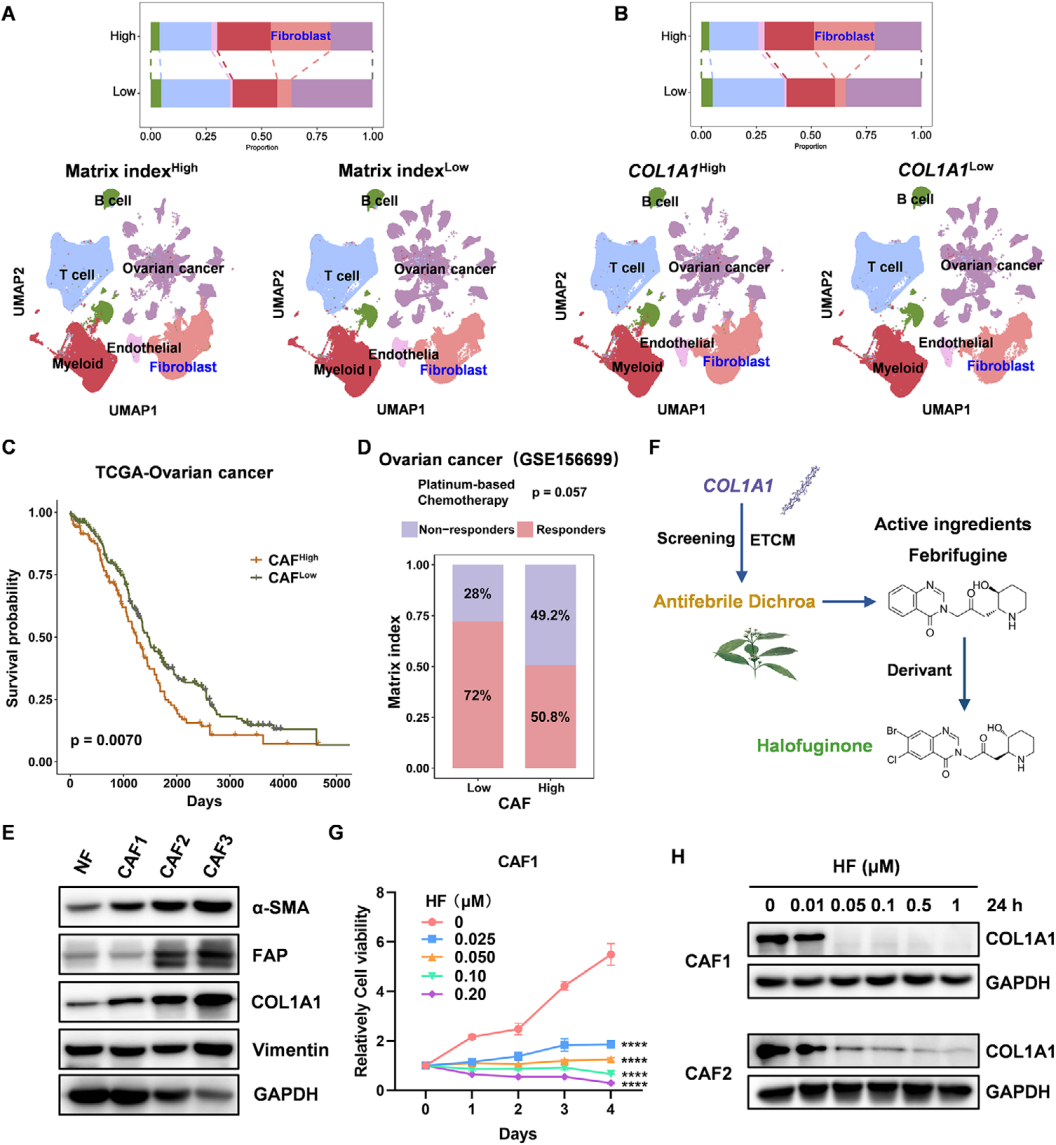
Halofuginone targets COL1A1 in CAFs. A,B) Different types of cell distributed in Matrix index^High^, Matrix index^Low^, COL1A1^High^, and COL1A1^Low^ groups using tSNE analysis from ovarian cancer scRNA‐seq data. C) Correlation between fibrosis and survival of ovarian cancer in TCGA dataset. D) Correlation between fibrosis and response to chemotherapy in ovarian cancer from GSE156699. E) Expression of Fibroblast‐associated markers in normal fibroblasts (NFs) and CAFs was determined by Western blot. F) Schematic illustration of halofuginone screening by ETCM. G) CAF1 was treated with different concentration of halofuginone as indicated, 24 h after treatment, and cell viability was detected by CCK8 assay. H) HF treated CAF1 and CAF2, respectively as indicated, after 24 h, COL1A1 expression was detected by Western blot. Statistical analysis was performed by two‐tailed, unpaired Student's t‐test and two‐way ANOVA with Tukey's test, ****p < 0.0001.

CAFs are the primary source of ECM components, including collagens, fibronectin, and matrix metalloproteinases 7 (MMP7), which create a pro‐invasive microenvironment to drive metastatic dissemination, modulate tumor stiffness, and facilitate tumor progression.^[^ ^34^, ^35^ ^]^ Based on ovarian cancer single cell RNA‐seq (scRNA‐seq) data, we observed that the proportion of fibroblasts was significantly higher in Matrix index^High^ or COL1A1^High^ group than in Matrix index^Low^ or COL1A1^Low^ group in ovarian cancer (**Figure** **3**A,B). Notably, patients with high fibrosis were associated with poor survival and chemotherapy response in ovarian cancer from TCGA dataset and GSE156699 (Figure 3C,D). Given to the heterogeneity of CAFs, we further analyzed different subtypes of CAFs between in Matrix index^High^ and Matrix index^Low^ groups. Based on expression of 21 CAF‐associated markers, we defined four subtypes of CAFs in ovarian cancer as follows: myofibroblast CAFs (myCAFs, 0: SMA+, 1: FAP+, 2: COL1A1+) and antigen‐presenting CAFs (apCAFs, 3: CD74+/HLA‐DRA+; Figure S4A,B, Supporting Information). The subtype of CAFs is a significant difference between Matrix index^High^ and Matrix index^Low^ groups. Tissues with Matrix index^High^ have more myCAFs, whereas tissues with Matrix index^Low^ have more apCAFs (Figure S4C,D, Supporting Information). As expected, myCAFs (subtypes 0 & 1) were markedly associated with chemoresistance in ovarian cancer, and apCAFs (subtype 3) were associated with chemosensitivity (Figure S4E, Supporting Information). These results indicate that CAFs mediate chemoresistance in ovarian cancer.

Collagen is predominantly produced by stromal cells, including CAFs and TAMs. We also found that COL1A1 expression is higher in fibroblasts compared to other subtype of cells using scRNA‐seq data (Figure S5, Supporting Information). To establish a desirable cell model to explore the strategies for targeting COL1A1, we isolated CAFs and NFs from freshly dissected human ovarian cancer tissues and adjacent normal ovarian tissues, respectively (Figure S6A, Supporting Information). As shown in Figure 3E and Figure S6B (Supporting Information), expression of α‐smooth muscle actin (α‐SMA), fibroblast activation protein (FAP), and COL1A1 is higher in CAFs than NFs by qRT‐PCR and Western blot, indicating that we successfully established ovarian cancer CAFs and NFs. To further ascertain whether COL1A1 influences additional malignant characteristics of CAFs, we established COL1A1 knockdown CAFs (Figure S7A, Supporting Information). Knockdown of COL1A1 in CAF2 can significantly inhibit ovarian cancer cell proliferation, migration, and invasion using a co‐culture model system (Figure S7B–I, Supporting Information). These findings suggest that disruption of COL1A1 might affect the function of ovarian cancer cells.

Given that COL1A1 may act as a potential therapeutic target in ovarian cancer. Collagen is produced by fibroblasts and surrounds the cancer cells, forming a protective physical barrier to block drug accumulation and promote chemoresistance.^[^ ^36^ ^]^ Therefore, targeting COL1A1 may sensitize cancer cells to chemotherapy. Natural compounds derived from animals, plants, or microorganisms are the safest and most effective way to treat human diseases. We screened the potential natural compounds of targeting COL1A1 using ETCM (http://www.tcmip.cn/ETCM), and found that antifebrile dichroa, a natural compound, emerged as a promising candidate (Figure 3F). HF, a derivative of febrifugine which is one of the active ingredients, is used for the prevention and treatment of coccidiosis and malaria in poultry.^[^ ^37^, ^38^, ^39^ ^]^ HF exhibited the excellent anti‐cancer, anti‐fibrosis, and great clinical potential for treating various cancers.^[^ ^40^ ^]^ Thus, we selected HF to explore its function for anti‐fibrosis.

To further assess whether HF inhibits CAFs‐secreted COL1A1, we treated ovarian cancer CAFs with HF and observed that HF could inhibit the viability of CAFs in a dose‐dependent manner (Figure 3G). While HF could significantly inhibit migration and invasion of CAFs (Figure S8, Supporting Information). More importantly, HF significantly inhibits CAF‐secreted COL1A1 in a dose‐dependent manner, even at 0.05 µm HF (Figure 3H). The previous study proved that tumor cells could specifically produce unique Collagen I homotrimers, thereby promoting pancreatic cancer progression.^[^ ^41^ ^]^ Consistent with this result, knockdown of COL1A1 may result in a reduction in the production and trimerization of COL1A1 using non‐denaturing gel electrophoresis and Western blot (Figure S9, Supporting Information). These results indicate that HF acts as a compound of targeting COL1A1 and inhibits CAFs‐secreted COL1A1.

### 2.4 Halofuginone Impairs Fibrosis by Reducing Collagen Deposition

**Figure 4:**
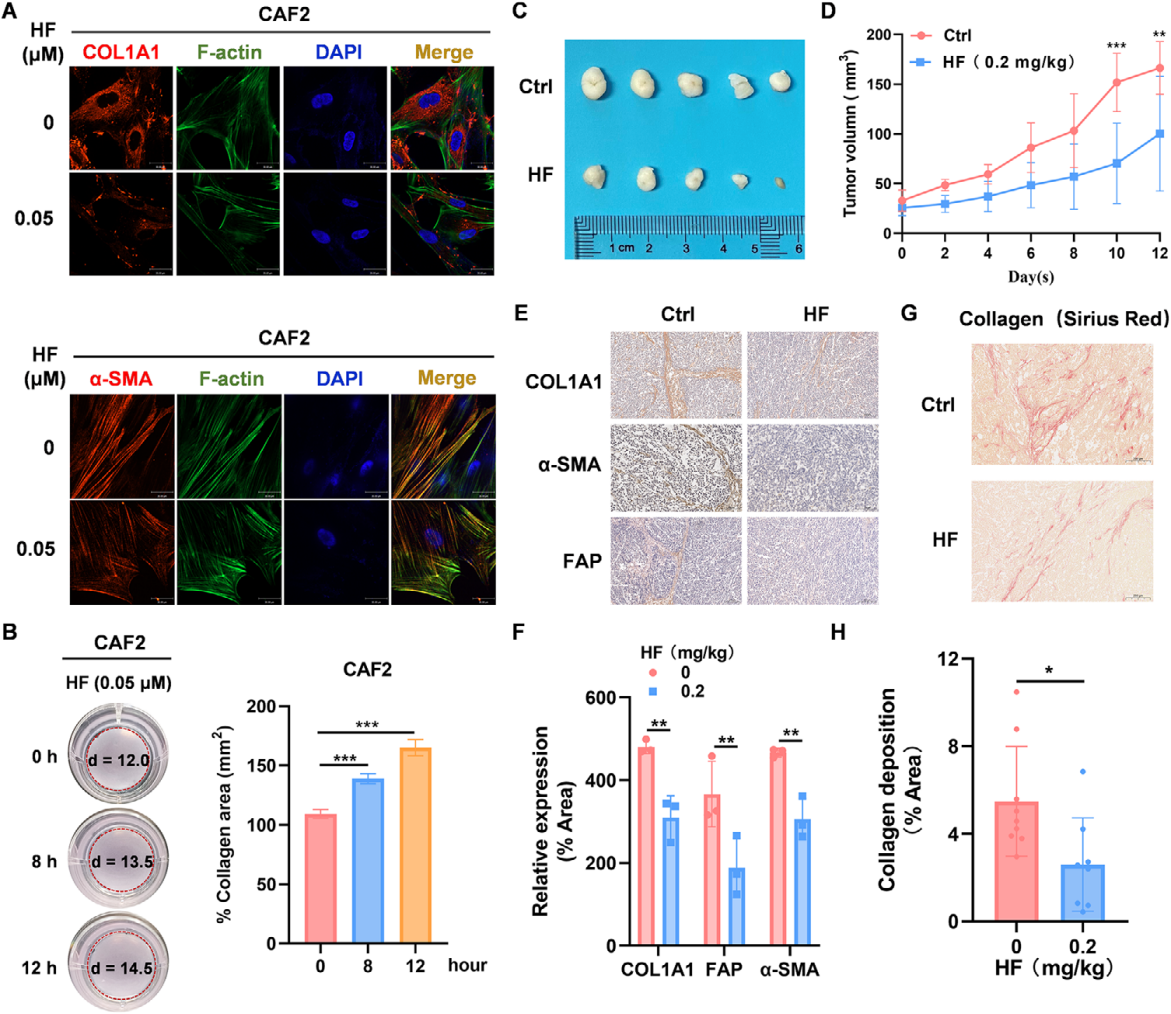
Halofuginone reduces collagen deposition to inhibit fibrosis. A) CAF2 was treated with 0.05 µm HF, 12 h after treatment, CAF‐specific markers: COL1A1 and α‐SMA (red), phalloidin‐stained cytoskeleton (green), and DAPI‐stained nuclei (blue) were detected by fluorescence microscopy. Scale bar, 30 µm. B) Quantification and representative images of gel contraction assay showing time‐dependent effect of HF on the ability of CAF2 to contract collagen in vitro. C,D) The therapeutic efficacy of HF was evaluated in ovarian cancer subcutaneous xenograft tumor model. C) Images of tumor tissues and D) tumor volumes, n = 5 mice per group. E,F) IHC assay of COL1A1, α‐SMA, and FAP expression in tumor‐bearing 3AO cells as indicated C. Scale bar, 200 µm. G,H) Sirius red staining of Col1 in tumor‐bearing 3AO cells as indicated C. Scale bar, 200 µm. Data was presented as mean ± SD (n = 3). Statistical analysis was performed by two‐tailed, unpaired Student's t‐test and two‐way ANOVA with Tukey's test, *p < 0.05, **p < 0.01, ***p < 0.001.

CAFs are the major stromal cells that produce ECM, and excessive production of fibrillar ECM proteins and ECM remodeling by CAFs in the TME leads to cancer fibrosis.^[^ ^17^ ^]^ Thereby, we examined CAF‐associated markers expression by immunofluorescence and observed that α‐SMA, FAP, and COL1A1 were significantly decreased in ovarian cancer CAFs under 0.05 µm HF treatment (**Figure** **4**A; Figure S10A, Supporting Information). Several different culture models were used to evaluate crosstalk between collagen‐mediated ECM and cancer cell in vitro. We observed that 0.05 µm HF could completely inhibit COL1A1 expression in CAF2 at 8 h after treatment (Figure S10B, Supporting Information), so we treated CAF2 with 0.05 µm HF for 8 h and 12 h, and found that HF inhibited collagen contraction using a cell contraction assay (Figure 4B). These results indicate that HF specifically inhibited collagen production and contraction in CAFs.

To further evaluate whether HF inhibits ovarian cancer cell growth through anti‐fibrosis, we used subcutaneous tumor models. Briefly, 3 × 10^6^ 3AO cells were subcutaneously inoculated into 5‐week‐old BALB/c nude mice, and the mice were randomly divided into two groups when the tumors reached ≈30 mm^3^. Subsequently, the mice were injected intraperitoneally with HF (0.2 mg k^−1^g) every two days for two weeks. Notably, HF could significantly restrain tumor growth, compared with control group (Figure 4C,D). Consistent with in vitro, HF could also significantly decrease expression of COL1A1, SMA, and FAP in vivo by IHC (Figure 4E,F), suggesting that HF could inhibit fibrosis. Fibrosis, defined as the excessive deposition of structural and matricellular proteins in the extracellular space, underlies tissue dysfunction.^[^ ^42^ ^]^ Therefore, to further assess interstitial fibrosis, we performed morphometric image analysis with Sirius Red and found that HF could significantly inhibit fibrosis in vivo (Figure 4G,H). Taken together, HF impairs fibrosis through inhibiting COL1A1 generation in ovarian cancer.

### 2.5 Halofuginone Inhibits COL1A1 Expression through mTOR‐eIF2α‐ATF4 Axis

**Figure 5:**
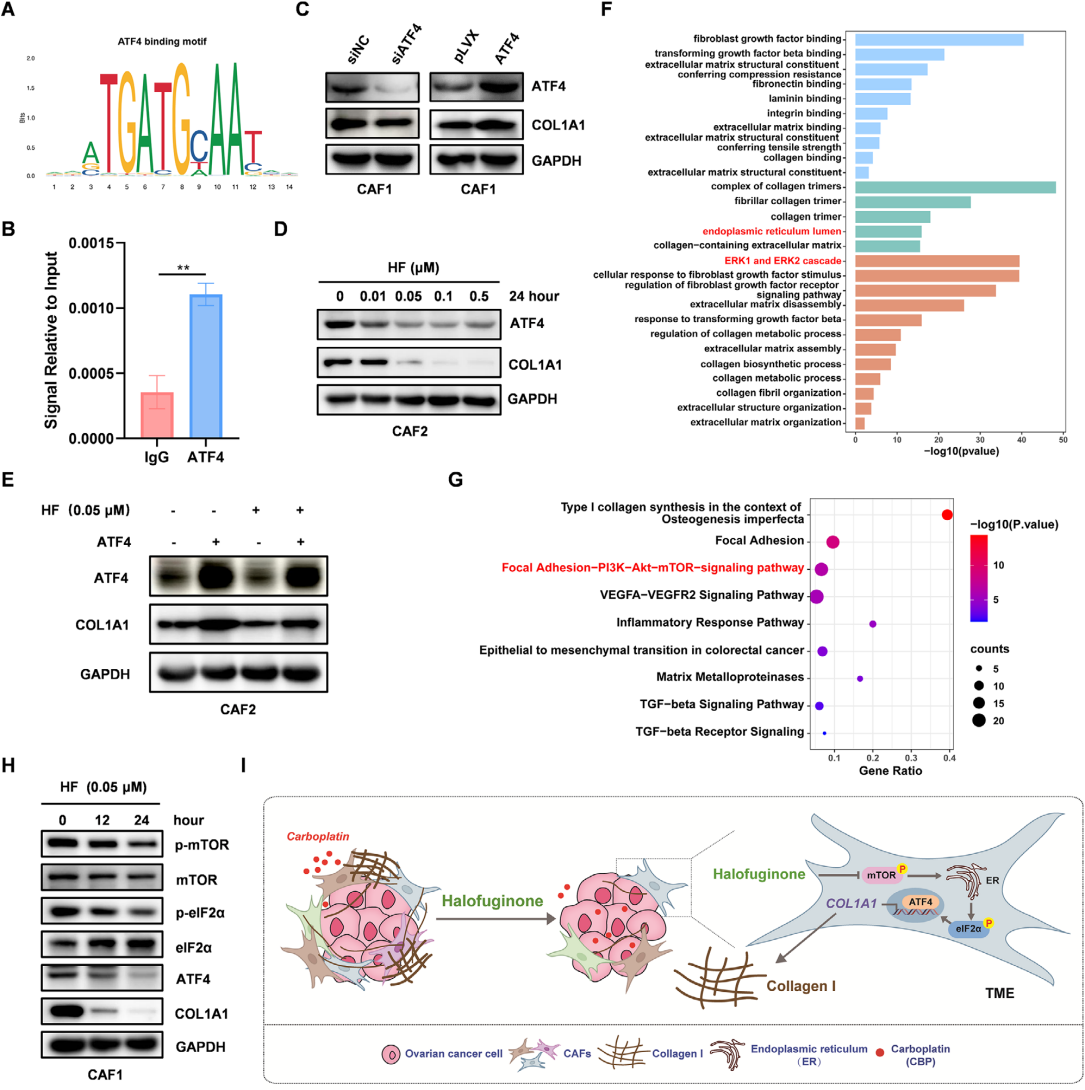
Halofuginone inhibits COL1A1 expression through mTOR‐eIF2α‐ATF4 axis. A) Analysis of ATF4 motif using JASPAR. B) ChIP‐qPCR was performed in CAF2 to identify the enrichment of ATF4 on the COL1A1 promoter region. IgG was used an antibody control. C) CAF1 was transiently transfected with ATF4 siRNA for 48 h and ATF4 plasmid for 24 h, respectively. Expression of ATF4 and COL1A1 was detected by Western blot. D) CAF2 was treated with different concentration of HF as indicated, ATF4 and COL1A1 expression was detected by Western blot. E) Western blot analysis of ATF4 and COL1A1 expression in CAF2 pretreated with 0.05 µm HF and then transfected with ATF4 as indicated. F,G) F) The bar chart and G) dot plot of GO and Kyoto Encyclopedia of Genes and Genomes (KEGG) pathway enrichment between COL1A1^High^ and COL1A^Low^ groups from public scRNA‐seq data of ovarian cancer, p < 0.05 is regarded as cut‐off value. H) CAF1 was treated with 0.05 µm HF, as indicated, and expression of mTOR‐eIF2α‐ATF4‐COL1A1 axis was detected by Western blot. I) Schematic illustration of mechanism by which HF inhibits COL1A1 expression and improves chemosensitivity through mTOR‐eIF2α‐ATF4 axis in ovarian cancer CAFs. Statistical analysis was performed by two‐tailed, unpaired Student's t‐test, **p < 0.01.

COL1A1 is regulated at the epigenetic, transcriptional, post‐transcriptional, and post‐translational levels including DNA methyl transferases (DNMTs), TGFβ, terminal nucleotidyltransferase 5A (TENT5A), or glucose‐regulated protein (Grp78).^[^ ^43^ ^]^ Previous studies have found that HF can inhibit the phosphorylation of STAT3, and p‐STAT3 may regulate transcription of COL1A1.^[^ ^44^, ^45^ ^]^ We also observed that HF could inhibit the expression of p‐STAT3 and the activation of TGFβ1/2 in CAF1 and CAF2 (Figure S11, Supporting Information). Stromal ATF4 as a key driver of CAF functionality directly regulates COL1A1 expression and collagen biosynthesis pathway.^[^ ^46^ ^]^ Similarly, ATF4 regulated the transcription of COL1A1 in ovarian cancer by ChIP‐qPCR (**Figure** **5**A,B). Inhibition of ATF4 could also decrease COL1A1 expression in CAF1, conversely, overexpression of ATF4 could also increase COL1A1 expression (Figure 5C). HF could decrease ATF4 expression in CAF2 (Figure 5D). ATF4 rescued COL1A1 expression under HF treatment in ovarian cancer CAF2 (Figure 5E). Notably, ATF4 was associated with COL1A1 in clinical ovarian cancer tissues from TCGA and GSE156699 datasets (Figure S12A,B, Supporting Information). ATF4 expression was associated with chemotherapy response and COL1A1 expression in ovarian cancer by IHC using tissues microarray (Figure S12C–E, Supporting Information). These results suggest that HF reduces COL1A1 expression through ATF4 in ovarian cancer CAFs.

To explore how HF regulates COL1A1 expression, we analyzed the pathway involved in COL1A1^High^ group in ovarian cancer scRNA‐seq, and found that extracellular regulated protein kinases (ERK) and endoplasmic reticulum pathway may be involved in regulating COL1A1 expression (Figure 5F). Previous studies have shown that ATF4 expression was regulated through ERK activation and eIF2α phosphorylation.^[^ ^47^ ^]^ HF can inhibit AKT/mTOR signaling pathway.^[^ ^48^ ^]^ Therefore, we performed the analysis of GO and KEGG pathway enrichment between COL1A1^High^ and COL1A^Low^ groups from public scRNA‐seq data of ovarian cancer, and found that AKT/mTOR signaling pathway might be involved in regulating progress of ECM (Figure 5G). Therefore, we speculated that HF might inhibit COL1A1 expression through mTOR‐eIF2α‐ATF4 axis. As expected, HF remarkably inhibited phosphorylation of mTOR and eIF2α in ovarian cancer CAF1 and CAF2 respectively, which in turn affected ATF4 expression in a time‐dependent manner (Figure 5H; Figure S13, Supporting Information). Collectively, these results indicate that HF inhibited COL1A1 expression through mTOR‐eIF2α‐ATF4 axis in ovarian cancer CAFs (Figure 5I).

### 2.6 Halofuginone Facilitates Immune Response to Sensitize Chemotherapy

**Figure 6:**
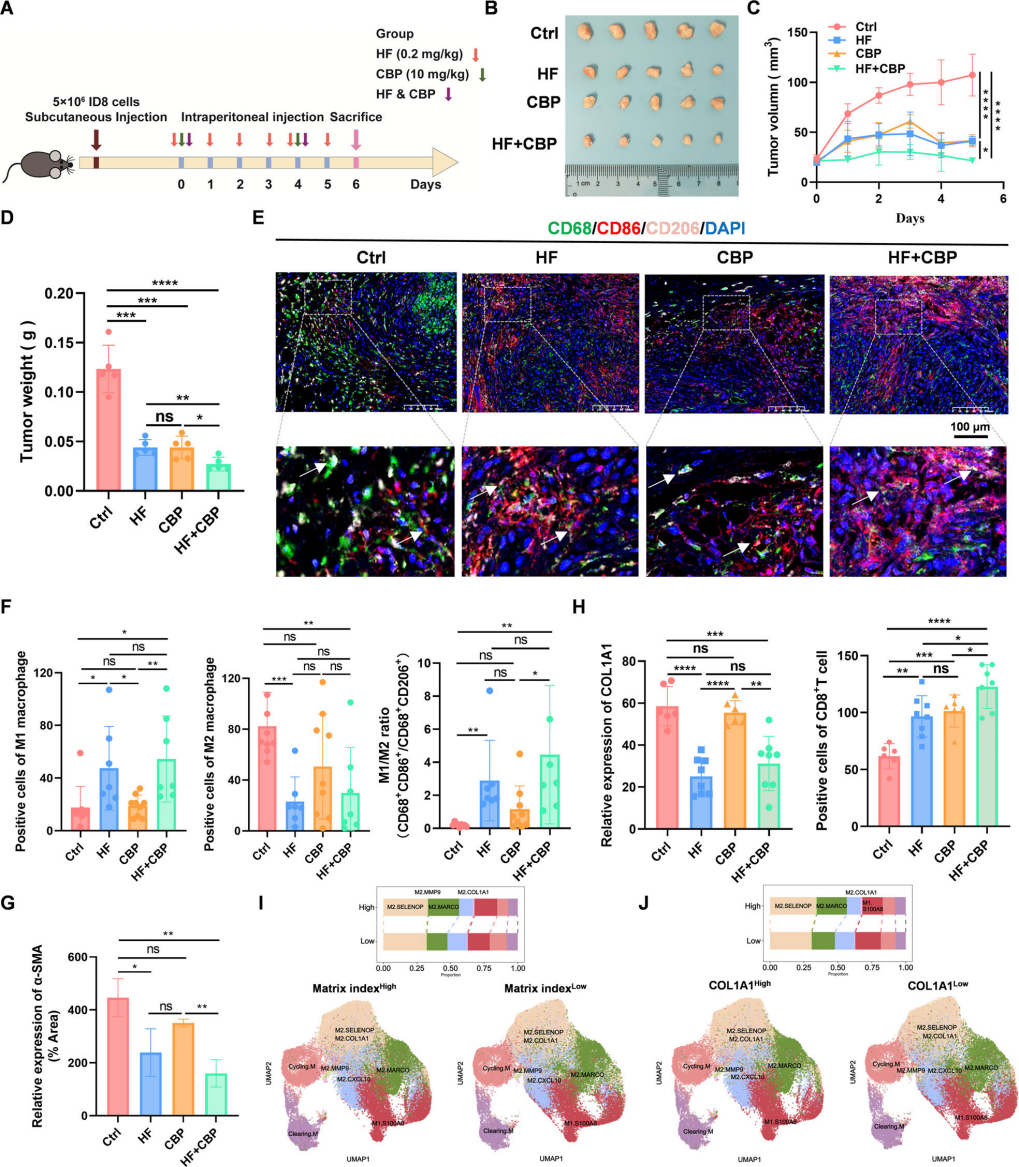
Halofuginone combined with Carboplatin reshapes TME to improve chemosensitivity. A) Schematic illustration of therapeutic experiments in subcutaneous tumor models. B–D) The therapeutic efficacy of HF, CBP, HF & CBP was shown in ovarian cancer subcutaneous tumor model. Images of B) tumor tissues, C) tumor volume, and D) tumor weight, n = 5 mice per group. E,F) The expression of CD68 (green), CD86 (red), and CD206 (pink) in these tissues as indicated Figure 6B was examined by multiplex immunohistochemistry (mIHC). E) The representative images in each group as indicated, scale bar, 100 µm, and F) the statistical analysis of these protein expressions. Data was presented as mean ± SD (n = 3). G) α‐SMA expression was examined by IHC. Data was presented as mean ± SD (n = 3). H) Analysis of COL1A1 and CD8+ T cells as indicated. Data was presented as mean ± SD (n = 3). I) The tSNE plots showing the percentage of TAMs type between Matrix index^High^ and Matrix index^Low^ groups from public scRNA‐seq data of ovarian cancer, p < 0.05 is regarded as cut‐off value. J) tSNE plots showing the percent of TAMs type in between COL1A1^High^ and COL1A^Low^ groups from public scRNA‐seq data of ovarian cancer, p < 0.05 is regarded as cut‐off value. Statistical analysis was performed by two‐tailed, unpaired Student's t‐test, *p < 0.05, **p < 0.01, ***p < 0.001, ****p < 0.0001.

Given that HF regulates the transcription of COL1A1 in vitro. To evaluate whether HF could influence deposition of collagen to facilitate immune cell infiltration in vivo, we first examined whether HF affected cell viability, and observed that HF could also significantly inhibit viability of mouse ovarian cancer cells ID8 in a dose‐dependent manner (Figure S14, Supporting Information). Subsequently, subcutaneous ovarian cancer model was used to evaluate the therapeutic efficacy of HF combined with CBP. Briefly, 5 × 10^6^ ID8 cells were subcutaneously inoculated into 5‐week‐old C57BL/6J female mice, and mice were randomly divided into four groups when the tumors reached ≈20 mm^3^. As shown in **Figure** **6**A, mice were intraperitoneally injected with Dimethyl sulfoxide (DMSO), 10 mg k^−1^g CBP, 0.2 mg k^−1^g HF, and HF & CBP. Notably, HF combined with CBP can significantly restrain tumor growth, compared with other groups (Figure 6B–D). To evaluate the potential toxicity of HF combined with CBP, we also performed hematological assessment and histological examination (H&E), and observed that body weight of C57BL/6J mice and blood biochemical indicators have no difference in all treatment groups (Figure S15A–D, Supporting Information). Additionally, normal morphology was observed in all treatment groups by H&E staining (Figure S15E, Supporting Information), demonstrating that HF combined with CBP was relatively safe.

Several recent studies have identified CAFs that drive a collagen ECM synthesis program in TAMs that is tightly regulated by TGFβ in breast cancer.^[^ ^24^ ^]^ Inflammatory monocytes and tissue‐resident macrophages are key regulators of tissue fibrosis, playing major roles in the initiation, maintenance, and resolution of tissue injury.^[^ ^49^ ^]^ Therefore, we set out to investigate whether ECM, especially collagen, limits antitumor immunity and responsiveness to chemotherapy. To explore TAMs in tissues after HF & CBP treatment, we performed mIHC to detect TAMs‐associated markers expression including CD68, CD86, and CD206. As shown in Figure 6E,F and Figure S16A (Supporting Information), we observed that HF significantly facilitated the conversion of M2 to M1 macrophages in vivo. Previous studies have shown that M2 macrophages repolarized to M1 macrophages might be activated and facilitated CD8+T cell infiltration.^[^ ^50^, ^51^ ^]^ In addition, HF could also significantly reduce expression of α‐SMA in tumor‐bearing ID8 cells by IHC (Figure 6G; Figure S16C, Supporting Information). HF could disrupt the physical barrier through inhibiting CAFs‐mediated COL1A1 production, thereby causing ECM to loosen from denseness. The looser ECM environment caused by HF is more conducive to CD8+ T cell infiltration (Figure 6H; Figure S16B, Supporting Information).

Given that HF altered the tumor immune microenvironment in vivo. The complex ECM remodeling processes may influence immune cell infiltration.^[^ ^52^ ^]^ TAMs can produce collagen and obstruct antitumor immunity not only by physically excluding CD8+ T cells, but also by inducing a mechano‐metabolic reprogramming of TAMs, resulting in an unfavorable metabolic landscape for CD8+ T cells.^[^ ^24^ ^]^ Based on ovarian cancer scRNA‐seq, we also observed that the ratio of M1/M2 macrophages is lower in patients with high Matrix index than in those with low Matrix index (Figure 6I). The ratio of M1/M2 macrophages is lower in patients with high COL1A1 expression than in patients with low COL1A1 expression (Figure 6J; Figure S17, Supporting Information). Overall, these results indicate that HF can inhibit COL1A1 production to reshape ECM and facilitate CD8+ T cell infiltration by partially increasing the ratio M1/M2 macrophages, thereby enhancing chemosensitivity.

## 3 Discussion and Conclusion

The interplay between ECM remodeling and cancer therapeutics opens up new avenues for understanding cancer biology and its effective treatment. CAFs secrete collagens and remodel ECM to form a physical barrier, hinder drug accumulation, and facilitate CD8+ T cell infiltration. Here, a 22‐gene matrisome signature or COL1A1 could predict chemotherapy response and prognosis in ovarian cancer. HF inhibited CAF‐mediated COL1A1 production through mTOR‐eIF2α‐ATF4 axis and facilitated CD8+ T cell infiltration by partially increasing M2‐M1 macrophage polarization, thereby enhancing chemosensitivity in vivo. Our findings indicate that HF combined with chemotherapy could be offered as a promising and effective cancer therapy.

ECM is a complex and dynamic network of ≈300 core proteins that constitutes the scaffold organizing cells, tissues, and organs.^[^ ^53^, ^54^ ^]^ ECM mainly exists in two main forms, interstitial matrix and basement membrane, where the interstitial matrix is composed of collagen, fibronectin, and elastin that connect cells to the stroma.^[^ ^14^ ^]^ Deposition of fibrillar collagen leads to stiffening of tumor tissue and promotes chemoresistance. Therefore, a comprehensive understanding of ECM properties would contribute to the discovery of promising therapeutic targets for cancer treatment. In our study, we identified that COL1A1, a core ECM protein, was associated with chemoresistance and poor prognosis, suggesting that COL1A1 is a potential therapeutic target in ovarian cancer. A previous study has shown that high COL1A1 expression was associated with poor survival and CBP‐resistance in ovarian cancer, and ZINC000085537017 and quercetin were potential drugs for COL1A1 based on virtual screening and the TCMSP database.^[^ ^55^ ^]^ Collectively, our study and the aforementioned research, both confirm the critical role of COL1A1 in chemoresistance from two different perspectives, thereby suggesting that the disruption of COL1A1 expression in cancer cells and/or stromal cells may represent an effective strategy for overcoming chemoresistance.

Therapeutic approaches intended to treat fibrosis in the TME, such as sonic hedgehog inhibition, have not yet yielded clinical success.^[^ ^56^ ^]^ To search for a promising collagen targeting natural drug, we identified HF using ETCM, as a synthetic derivative of the natural product febrifugine isolated from an ancient herbal remedy. HF and febrifugine derivatives have been used to treat malaria, cancer, fibrosis, and inflammatory diseases. As a prolyl‐tRNA synthetase (PRS) and TGFβ inhibitor, HF suppresses fibrosis by regulating TGFβ signaling pathway, reducing collagen product, decreasing ECM proteins, and preventing TH17 differentiation.^[^ ^26^, ^57^, ^58^, ^59^, ^60^, ^61^ ^]^ Consistent with these results, HF can also inhibit the activity of TGFβ1/2. Notably, we demonstrated that COL1A1 expression is regulated by mTOR‐eIF2α‐ATF4 axis in ovarian cancer CAFs. In addition, HF can also inhibit cell viability through enhancing CBP‐induced apoptosis in vitro (unpublished data). Our findings indicate that HF exhibits a dual inhibitory effect on cancer cells and CAFs. HF is a broad‐spectrum anti‐parasitic agent that is used as a feed additive to control coccidiosis in poultry,^[^ ^37^, ^62^, ^63^, ^64^ ^]^ indicating that HF is relatively safe and has the potential for clinical translation. However, HF has no tissue‐specificity and can target COL1A1 in fibroblasts, as well as exert effects on normal tissues. Therefore, the precise targeting of COL1A1 produced by CAFs rather than normal tissues will be the subject of further investigation.

Emerging studies showed that various cancer therapies induce ECM remodeling, resulting in therapeutic resistance and tumor progression.^[^ ^14^, ^29^ ^]^ Targeting collagen biosynthesis and cross‐linking could improve therapeutic efficacy. CAF‐secreted collagen XII alters collagen I organization and creates a pro‐invasive microenvironment to drive metastatic dissemination.^[^ ^65^ ^]^ Targeting lipoxygenases (LOX) by β‐aminopropionitrile (BAPN) overcomes chemoresistance in triple‐negative breast cancer.^[^ ^66^ ^]^ Here, our findings demonstrated that HF could significantly inhibit ovarian cancer cell growth in vivo. Recent studies showed that CAF drives a collagen ECM synthesis program in TAMs that is tightly regulated by TGFβ in breast cancer.^[^ ^24^ ^]^ HF enhances the efficacy of CD8+ T cells in models of adoptive cell therapy.^[^ ^67^ ^]^ In the subcutaneous graft ovarian cancer model, HF can enhance chemosensitivity in part by disrupting the physical barrier, facilitating CD8+ T cell infiltration partially via M2‐M1 macrophage polarization. Matrix index^High^ group has more TAMs infiltration in ovarian cancer based on scRNA‐seq. Our findings suggest that inhibition of collagen production by HF may improve tumor immune microenvironment. Nevertheless, one of the outstanding issues in our research is to ascertain why the physical characteristics of the ECM affect the conversion of macrophages from the M1 to the M2 phenotype. Another unresolved question is why the physical characteristics of the ECM influence the activation of CD8+ T cells. The potential of HF combined with immune checkpoint inhibitors (ICBs) in the treatment of ovarian cancer will be further investigated. Overall, our findings indicate that HF combined with chemotherapy may be a promising and effective approach for ovarian cancer treatment.

## 4 Experimental Section

### Cell Culture

The ovarian cancer cell lines SKOV3, TOV‐21G, A2780, and HEK293T cells were purchased from the American Type Culture Collection (ATCC; Manassas, VA, USA). TOV‐21G and SKOV3 cells were cultured in RPMI1640 medium supplemented with 10% fetal bovine serum (FBS; Cell Technologies, Beijing, China), 1% penicillin and streptomycin (PS; Cell Technologies, Beijing, China). A2780 and HEK293T cells were cultured in DMEM medium supplemented with 10% FBS, 1% PS. All cells were maintained at 37 °C in a humidified cell incubator containing 5% CO_2_.

### Tissue Microarray

A tissue microarray containing 119 ovarian cancer tissues (duplicate 2.2 mm tissue cores for each sample) was constructed by Li΄s laboratory.^[^ ^68^ ^]^ These patients were accepted cytoreductive surgery and platinum‐based chemotherapy from May 2009 to January 2013 at the Department of Gynecologic Oncology, Cancer Hospital Chinese Academy of Medical Sciences. This study was approved by the Institutional Review Board of the Cancer Hospital Chinese Academy of Medical Sciences.

### Isolation of Human NFs and CAFs

Primary CAFs and NFs were isolated from fresh surgically resected ovarian cancer tissues and adjacent normal tissues. Within 1 h after surgery, tissues were digested in 1 mg mL⁻^1^ collagenase (Sigma‐Aldrich, St. Louis, MO) diluted in DMEM medium at 37 °C for 15 min with shaking. After centrifugation, the supernatant was discarded. The tissue blocks were seeded into cell culture flasks with DMEM/F12 medium (Thermo Fisher Scientific Inc, Shanghai, China) containing B27 (1:50; Invitrogen, Carlsbad, USA), 20 ng mL⁻^1^ Epidermal growth factor (EGF) (Sigma‐Aldrich, St. Louis, MO), 10 ng mL⁻^1^ Basic Fibroblast Growth Factor (bFGF) (Invitrogen, Carlsbad, USA), and 10 ng mL⁻^1^ TGFβ1 (MCE, Shanghai, China), and cultured for a week until fibroblasts crawled out of the tissue blocks. After two passages, primary CAFs and NFs were cultured in DMEM with 10% FBS.

### Western Blot

Cell pellets of denaturing gel electrophoresis were collected as indicated and lysed in RIPA lysis buffer (NCM Biotech, Suzhou, China) containing protease and phosphatase inhibitors. Cell pellets of non‐denaturing gel electrophoresis were collected as indicated and lysed with IP buffer (50 mm Tris‐Cl, 150 mm NaCl, 1 mm EDTA, 1% Triton X‐100). Proteins were quantified using an Enhanced BCA Protein Assay Kit (Thermo Fisher Scientific Inc). A western blot was performed according to standard protocols, and GAPDH was used as an endogenous control. The antibodies used in this study are listed in Table S2 (Supporting Information).

### RNA Extraction and RT‐qPCR

Total RNA was extracted from cells as indicated using TRIzol reagent (Invitrogen), and then reverse transcribed into cDNA using a FastKing RT Kit (TIANGEN, Beijing, China). qRT‐PCR was performed on a StepOnePlus Real‐Time PCR system using standard procedures. The relative expression levels of CAF‐associated genes were normalized to GAPDH, which served as an endogenous control. Primers are listed in Table S3 (Supporting Information).

### Collagen Gel Contraction Assay

The ability of collagen contraction was detected by using a cell contraction assay kit (Cell Biolabs Inc, San Diego, USA). CAFs were seeded in 24‐well plates in DMEM with 10% FBS, and collagen gel working solution at a density of 4 × 10^6^ cells/mL. After 2 days, treated with 0.05 µm HF for different times (0, 8, and 12 h). The contractions were calculated as collagen area (mm^2^).

### Chromatin Immunoprecipitation (ChIP) Assays

ChIP assays were performed using SimpleChIP Enzymatic Chromatin IP Kit (Cell Signaling Technology, Shanghai, China) according to the manufacturer's instructions. 37% formaldehyde was added to the medium for crosslinking proteins with DNA, and then 1.25 m glycine was used to terminate this reaction. The crosslinked cells were digested and sonicated to achieve DNA fragments in a size ranging from 150 to 900 bp. The sonicated chromatin was incubated with ATF4 (Cell Signaling Technology, Shanghai, China) or IgG antibody for overnight at 4 °C, and then added ChIP‐Grade Protein G Magnetic Beads for 6 h at 4 °C. DNA was separated from immunoprecipitated mixture using thermomixer and purified with ChIP assay kit. Finally, qPCR was applied to examine the ATF4 enrichment on the regulatory region of COL1A1. Primers for ChIP‐qPCR were listed in Table S4 (Supporting Information).

### ELISA Assay

Protein concentration levels of TGFβ1 and TGFβ2 were determined in conditioned medium of CAF1 and CAF2 treated with 0.05 µm HF for 24 h using ELISA according to the manufacturer's instructions (LiankeBio, Hangzhou, China). Absorbance at 450 nm was recorded using a micro‐plate reader (Thermo Fisher Scientific Inc, Shanghai, China).

### mIHC

From IHC analysis, the sections obtained from the subcutaneous tumors treated with DMSO, HF, CBP, and HF & CBP were hybridized with antibodies as indicated according to the manufacturer's protocol of multiplex IHC Detection kit (Aifang biological, Hunan, China). All images were batch‐analyzed using K‐viewer automated image analysis software (KFBIO, Zhejiang, China).

### Animal Models

For the subcutaneous transplantation models, 3 × 10^6^ 3AO cells were subcutaneously injected into 5‐week‐old female BALB/c nude mice. When the tumors reached ≈30 mm^3^, the mice were randomly divided into two groups as indicated and treated with DMSO or HF (0.2 mg k^−1^g) every two days for two weeks by intraperitoneal injection. Tumor sizes were measured every two days. For therapeutic models, 5 × 10^6^ ID8 cells were subcutaneously injected into 5‐week‐old female C57BL/6J mice. When the tumors reached ≈20 mm^3^ in volume, the mice were randomly divided into different groups as indicated and treated with DMSO, CBP (10 mg k^−1^g), HF (0.2 mg k^−1^g), and HF & CBP by intraperitoneal injection. HF was injected every day for six times, and CBP was injected every 4 days for two times. Tumor sizes were measured every day. A week later, all the mice were euthanized. Tumors were harvested and subjected to paraffin‐embedded sections for immunohistochemical staining. n = 5 mice per group; no animals were excluded. All animal protocols were approved by the Animal Care and Use Committee of the Chinese Academy of Medical Sciences Cancer Hospital.

### Matrix Index and Fibrosis Assay

The transcript levels of a 22‐gene set of matrisome that could predict both disease extent and tissue stiffness in different tumor sample sets to construct a Matrix index using the single‐sample gene set enrichment analysis (ssGSEA) method were utilized. The ovarian cancer scRNA‐seq data were downloaded from Synapse (https://www.synapse.org/msk_spectrum, accession number syn25569736). The average COL1A1 expression across all cells for each sample was calculated to determine the overall COL1A1 expression level in each sample. Subsequentially, these samples were divided into COL1A1^High^ and COL1A1^Low^ groups based on the median expression level. Fibroblasts were divided into COL1A1^High^ and COL1A1^Low^ groups based on the median expression of COL1A1. DEGs between these two groups were then identified using the FindMarkers function from the Seurat package. GO and KEGG analyses were then performed using the clusterProfiler package.

### Statistical Analysis

Data analysis was performed using GraphPad Prism (Version 9.5.1.7), using One/Two‐way ANOVA, two‐tailed, and unpaired Student's t‐test. All statistical results represent the mean± SD. Pearson's correlation was used to analyze the correlation between ATF4 and COL1A1 expression. The Kaplan‐Meier method and the log‐rank test were used to assess survival. The efficacy of chemotherapy was analyzed by Fisher's exact test. For all comparisons, p < 0.05 was considered statistically significant.

## Conflict of Interest

The authors declare no conflict of interest.

## Author Contributions

A.L. and Z.L. assisted in concept and design; Y.W., Y.Z, and W.G. assisted in data collection, analysis, and interpretation; A.L., Z.L., and W.L. assisted in writing, reviewing, and/or revising manuscripts; X.L., H.L., and M.L. assisted in administrative, technical, or material support.

## Supporting information



Supporting Information

Supporting Information

Supporting Information

Supporting Information

Supporting Information

Supporting Information

## Data Availability

The data that support the findings of this study are available from the corresponding author upon reasonable request.
