# Supplementary material for: Halofuginone Disrupted Collagen Deposition via mTOR‐eIF2α‐ATF4 Axis to Enhance Chemosensitivity in Ovarian Cancer
Source: Adv Sci (Weinh). 2025 Mar 24;12(19):2416523. doi: 10.1002/advs.202416523 (PMC12097005; doi:10.1002/advs.202416523)
Supplement: Supplementary file 1 — Supporting Information [file ADVS-12-2416523-s003.docx]

Supporting information

**Halofuginone Disrupted Collagen deposition via** **mTOR-eIF2α-ATF4 axis to** **Enhance Chemosensitivity in Ovarian Cancer**

*Wenxin Li, Yenan Wu, Yanan Zhang, Wenyan Gao, Xin Li，Haixia Luo，Mengmeng Lu, Zhihua Liu^*^, Aiping Luo^*^*

**Supporting information methods**

The reagents and kits used in this study were listed in Table S5 (Supporting Information).

*Immunohistochemistry*: For immunohistochemistry (IHC) analysis, ovarian cancer tissue microarrays and the sections obtained from the subcutaneous tumors treated with HF or not were hybridized with antibodies as indicated according to the manufacturer’s protocol. After hybridization, the sections were scanned and imaged by a single investigator who was blinded to the clinical characteristics. The integral intensity value was measured using Aperio’s ImageScope software (Aperio, Vista, CA, USA).

*Immunofluorescence*: Cells were seeded into 6-well plates with slides. Briefly, after treatment as indicated, these slides were fixed with 4% paraformaldehyde (Servicebio, Hubei, China) for 1 h and permeabilized by 0.5% Triton X-100 (Aladdin, Shanghai, China) for 15 min at room temperature (RT). Slides were washed with 1× PBS, blocked with 5% BSA (Absin, Shanghai, China) for 1 h, and then incubated with antibodies as indicated for overnight at 4 ℃. Moreover, Phalloidin (Invitrogen, Carlsbad, USA) was used to stain cytoskeleton for 30 min at RT according to the manufacturer’s protocol. Cell nuclei were stained with DAPI (Invitrogen) for 15 min at RT. The images were captured with a fluorescence microscope.

*Cell viability assay*: For cell viability assay, 8×10^3^ SKOV3 and TOV-21G cells were seeded into 96-well plates coated with collagen type I (100 μg mL^-1^) or not as indicated. After HF treatment for 24 h, CCK-8 reagent (TargetMol, Shanghai, China) was added to the 1 × PBS at a ratio of 1:10, and the absorbance was measured using a microplate reader (BioTek, VT, USA) at 450 nm after incubation for 1 h at 37 ℃.

*Wound healing assay*: 1.5 × 10^4^ CAF1 were seeded into 96-well plates, next day, and CAF1 were allowed to reach 90%-95% confluence. A scratch was made in the center of each well, and the wells were washed twice with 1 × PBS to eliminate the interference of cell debris. 100 μL DMEM without FBS was added to each well and cultured in the incubator. The images were captured by microscope every 12 h and the difference in migration rate of CAF1 was observed.

*Cell migration and invasion*: 4×10^4^ CAF1 was resuspended in 100 μl FBS-free DMEM, seeded in the upper chamber with or without Matrigel (Corning, MA, USA), and then 650 μl DMEM with 10% FBS were added to the lower chamber. After 24 h, the upper chamber was placed in crystal violet and stained for 2 h at RT. After staining, the images were captured by microscopy and analyzed.

*Flow cytometry*: Apoptosis was detected by staining with annexin V-FITC using an apoptosis detection kit (Multisciences Co., Ltd, Zhejiang, China) according to the manufacturer’s protocol. Briefly, (1-10) ×10^5^ cells were harvested and resuspended in 400 μl mixing solution of 1 × binding buffer and annexin V (100:1). Subsequently, 8 μl propidium iodide (PI) was added, incubated for 5 min at RT in the dark, and detected by flow cytometry.

*Safety Evaluation*: Healthy C57BL/6J mice were injected as indicated. AT the end of treatment, blood was collected for the biochemical index evaluation, and the major organs of heart, liver, spleen, kidney, and lung were collected for standard hematoxylin and eosin (H&E) staining by Wuhan Servicebio Technology CO.,LTD.

**Supporting information figures**

**
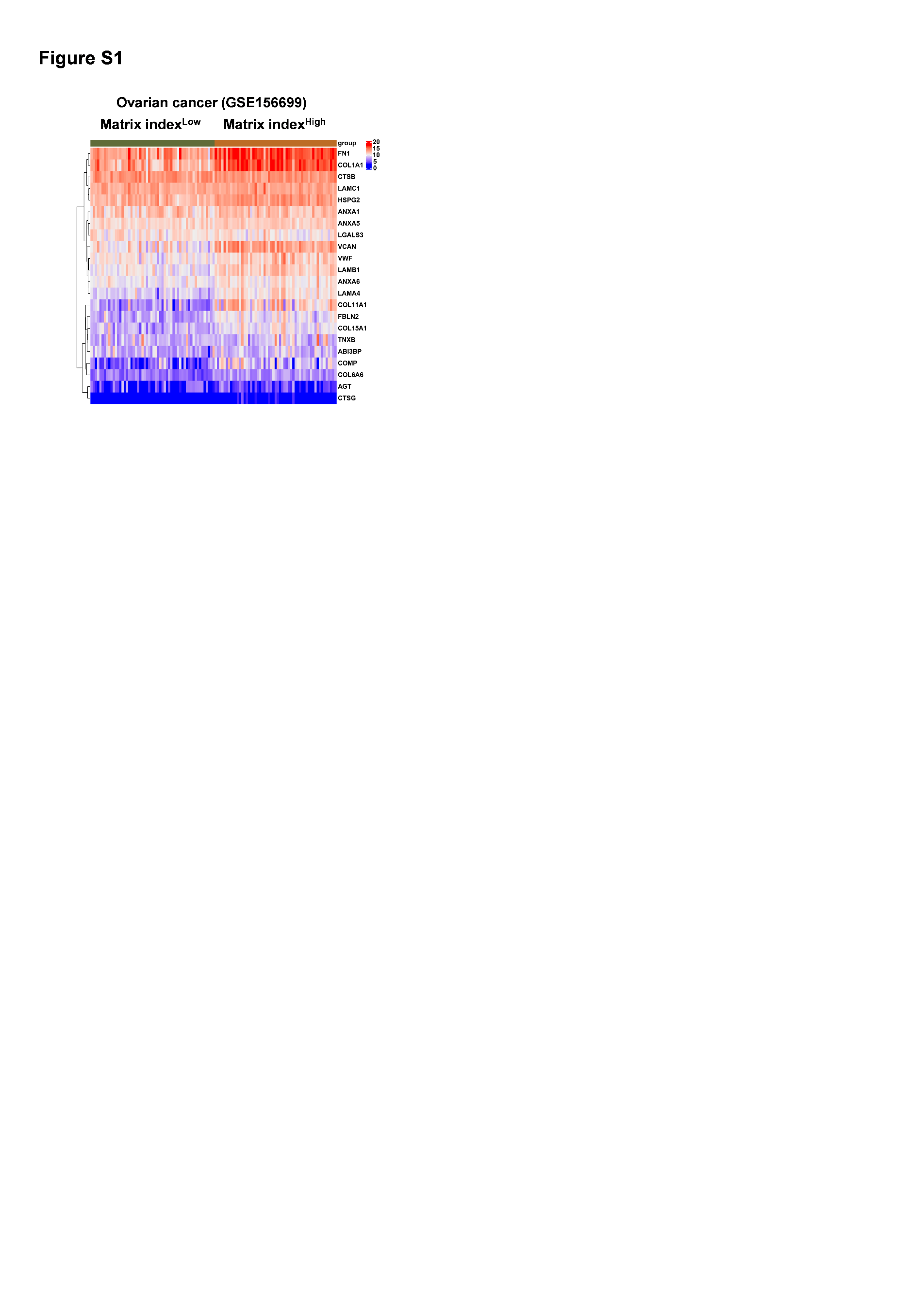
**

**Figure S1.** 22 matrisome genes expression in ovarian cancer. Ovarian cancer patients were divided into Matrix index^High^ group and Matrix index^Low^ group based on a 22-gene matrisome signature using GSE156699 dataset.

**
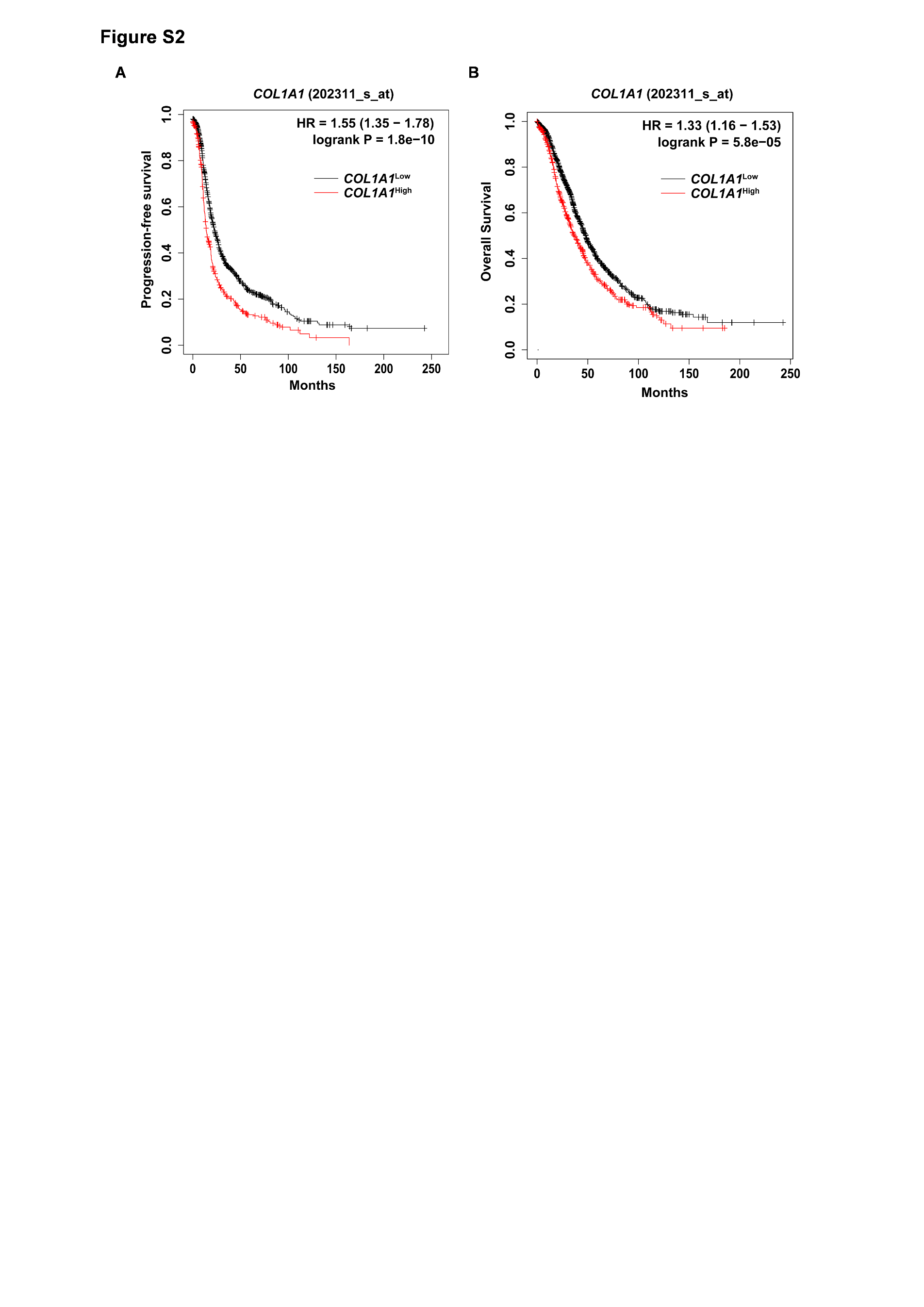
**

**Figure S2.** COL1A1 expression is closely associated with poor prognosis in ovarian cancer. A-B) Correlation between COL1A1 and overall survival (OS) or progression-free survival (PFS) in ovarian cancer using Kaplan–Meier analysis.


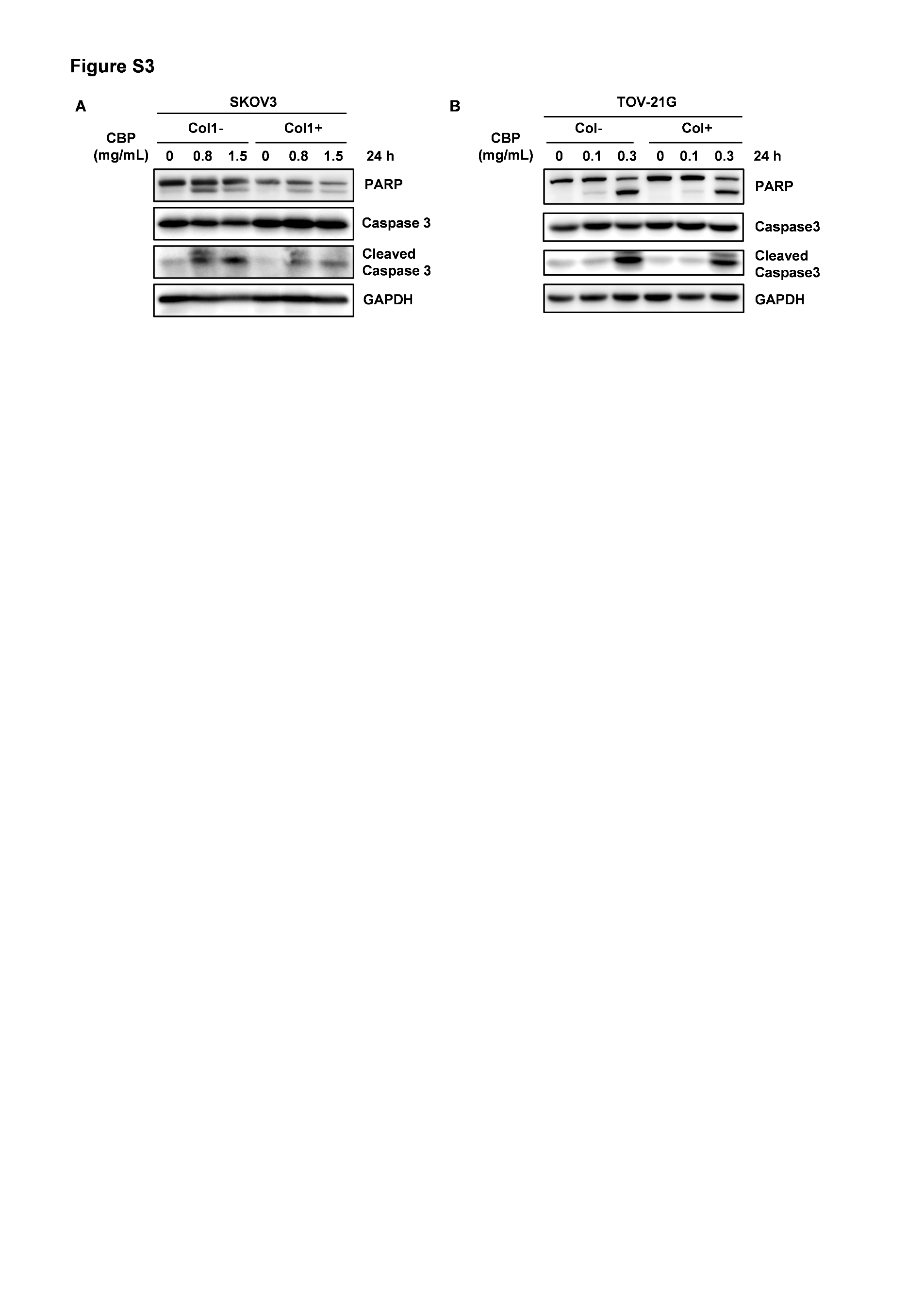


**Figure S3.** Collagen resists carboplatin treatment. A-B) SKOV3 and TOV-21G cells were seeded in 6-well plates precoated with Col1 (100 μg mL^-1^) or without, and then treated with CBP, 24 h after treatment, expression of apoptosis-associated markers was examined by Western blot.


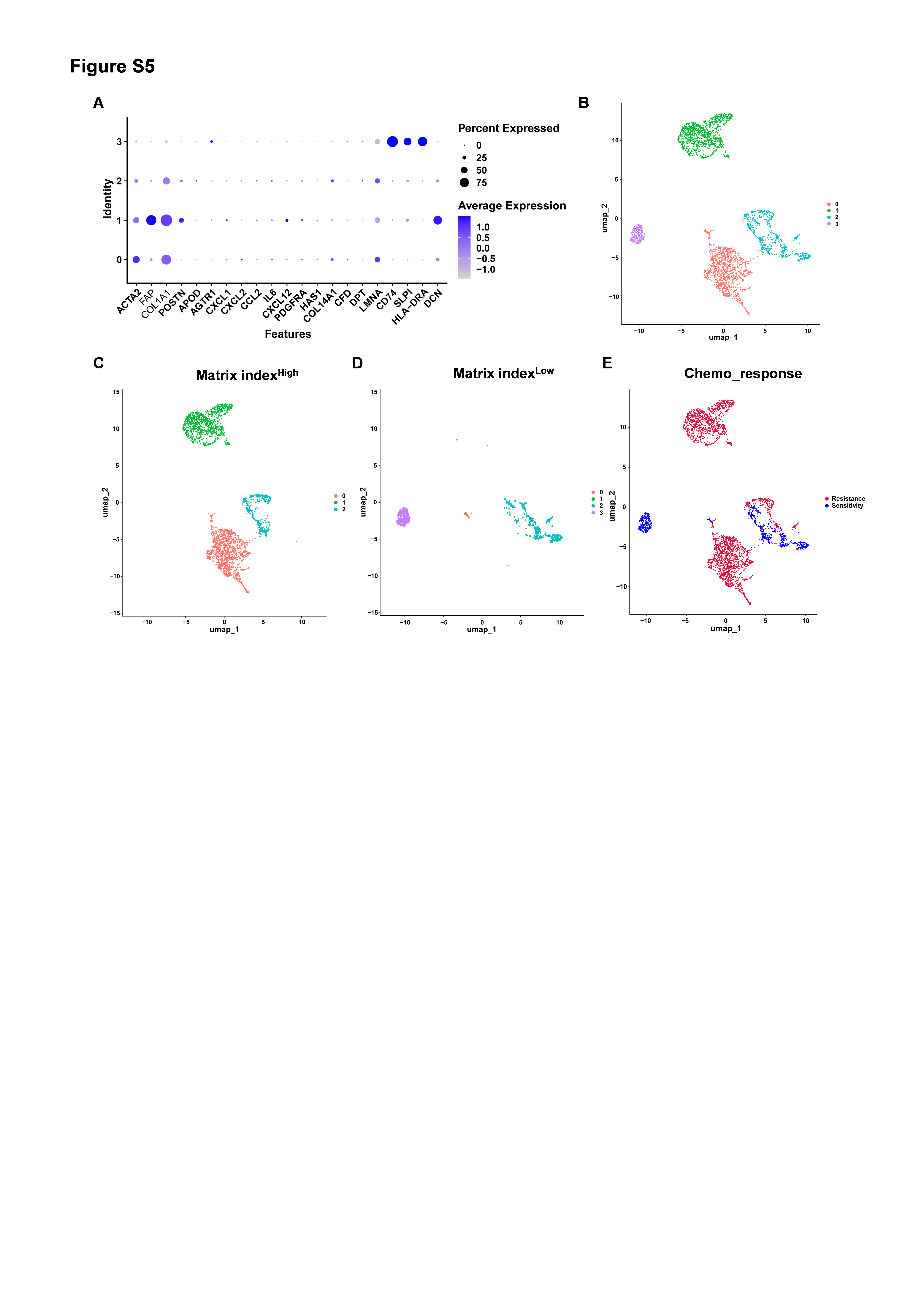


**Figure S4.** The subtype of CAF associated with chemoresistance. A) Expression of 21 CAF-associated markers in subtype of CAFs; B) 4 subtypes of ovarian cancer CAFs; C-D) The difference of subtype of CAFs between in Matrix index^High^ and Matrix index^Low^ groups; E) The difference of subtype of CAFs between in resistance and sensitivity group in ovarian cancer.


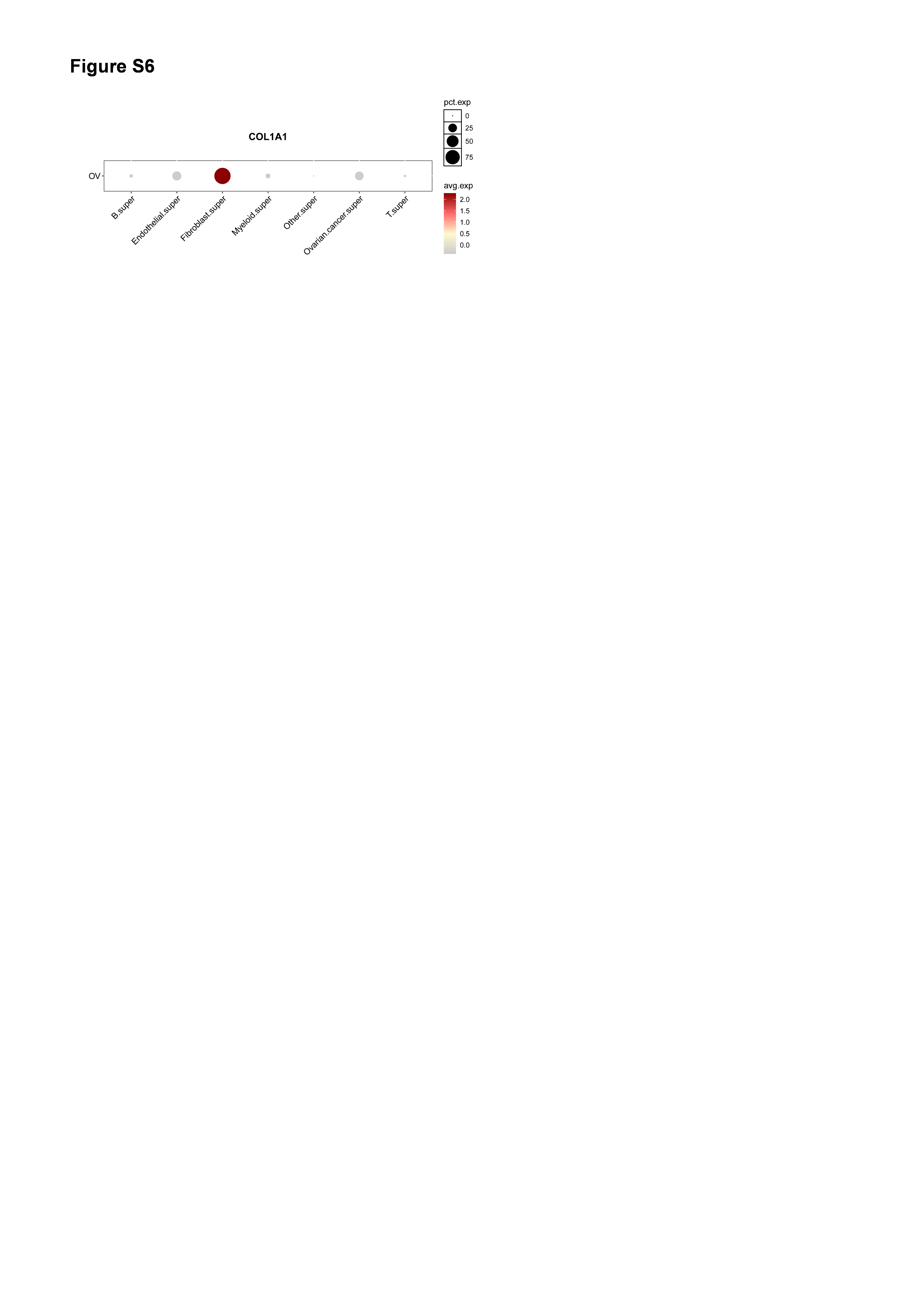


**Figure S5.** COL1A1 expression in different subtype of cells. Different types of cells between COL1A1^High^ and COL1A1^Low^ groups from public ovarian cancer scRNA-seq data.


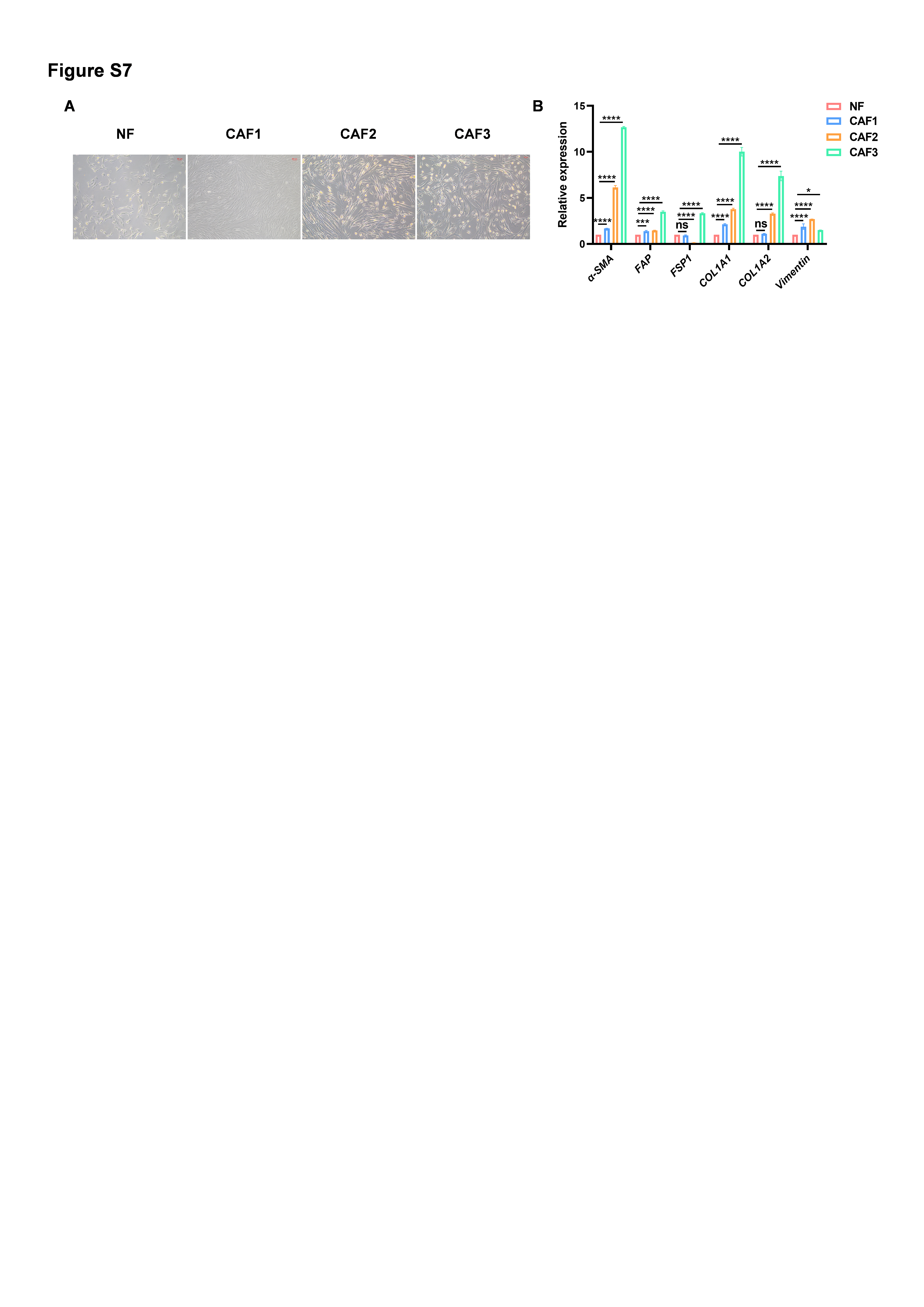


**Figure S6.** Isolation and identification of NFs and CAFs. A) Isolated CAFs and NFs from freshly dissected human ovarian cancer tissues and adjacent normal ovarian tissues, respectively. Representative images for NFs and CAFs by microscope. B) Expression of Fibroblast-associated markers in NFs and CAFs was determined by qRT-PCR.


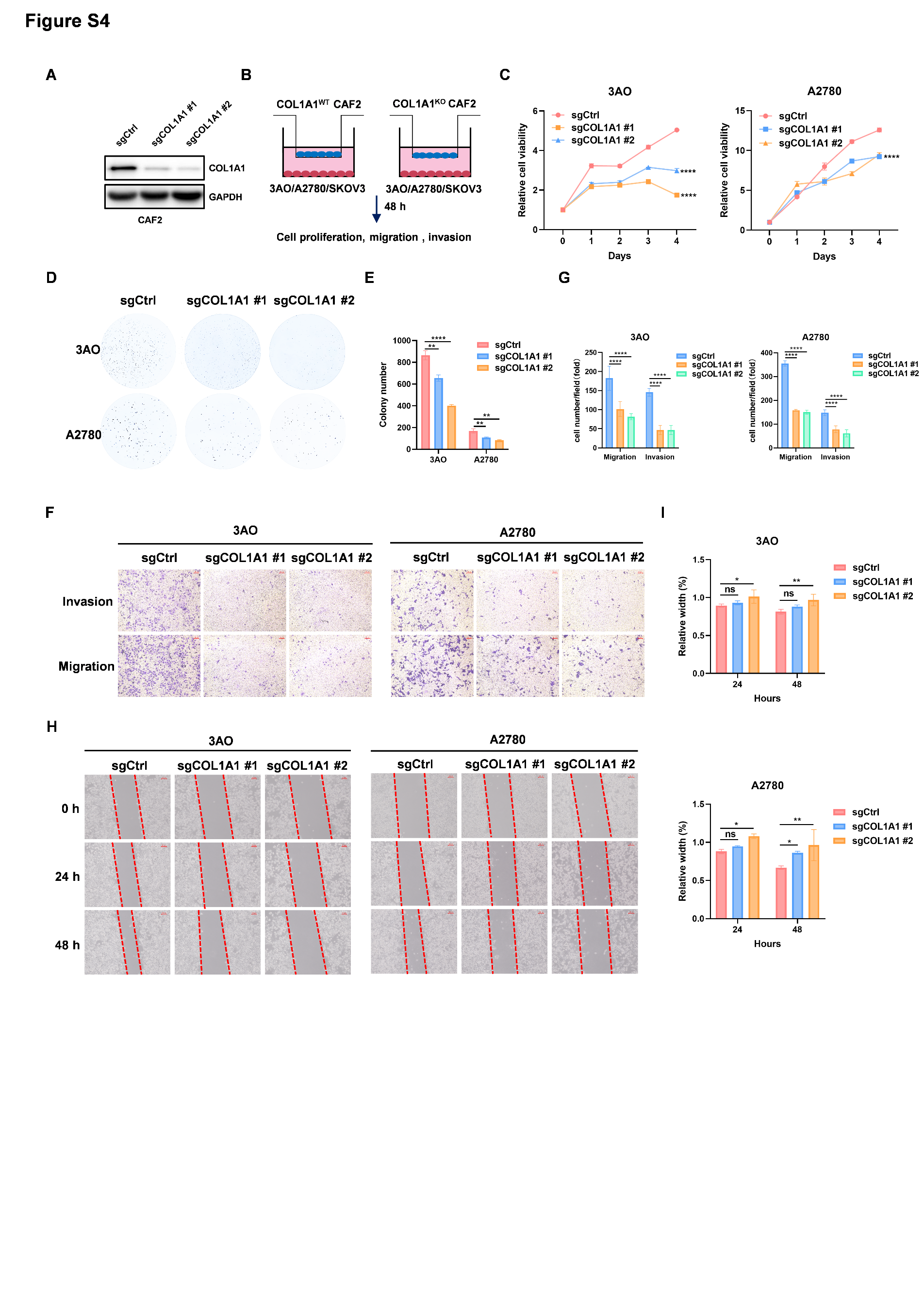


**Figure S7.** COL1A1 knockout CAF inhibited the proliferation, invasion, and migration of ovarian cancer cells. A) COL1A1 expression of COL1A1 knockout CAF by western blot. B) Schematic of COL1A1 knockout CAF-ovarian cancer cells co-culture model system. C-D) After co-culture for 48 h, the cell proliferation was detected by colony formation assay. E) After co-culture for 48 h, the cell proliferation was detected by CCK8 assay. F-G) After co-culture for 48 h, the cell migration and invasion were assessed by Transwell assay. H-I) COL1A1 was knocked in CAF2. After 24 hours，the CAF2 co-cultured with 3AO and A2780 cells, respectively. After 48 hours, ovarian cancer cell migration was assessed by wound healing assay.


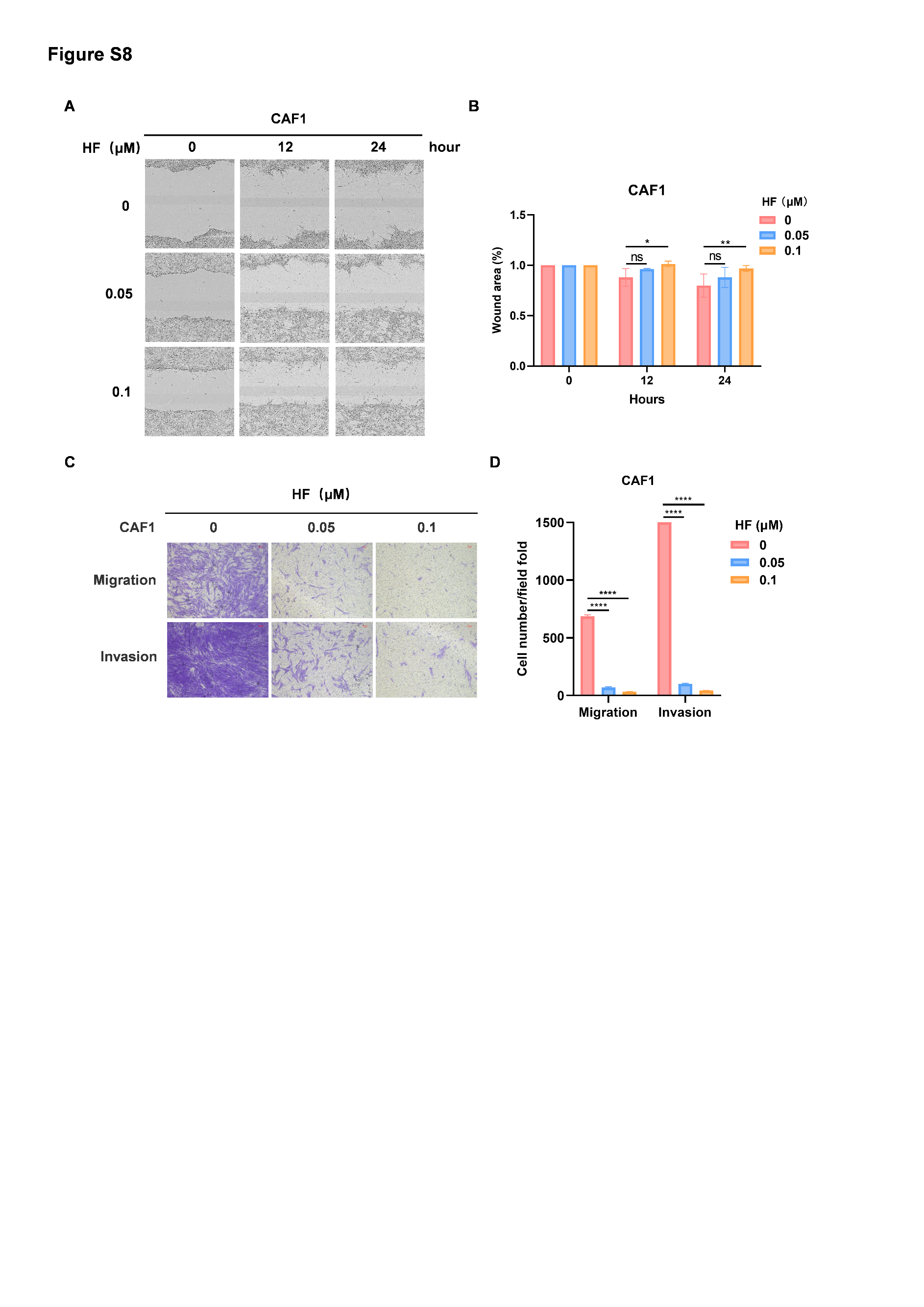


**Figure S8.** Halofuginone inhibits CAFs migration and invasion. A-B) CAF1 was treated with different concentration of HF as indicated, and cell migration was assessed by wound healing assay. C-D) CAF1 was treated with different concentration of HF as indicated, and cell migration and invasion were assessed by Transwell assay.


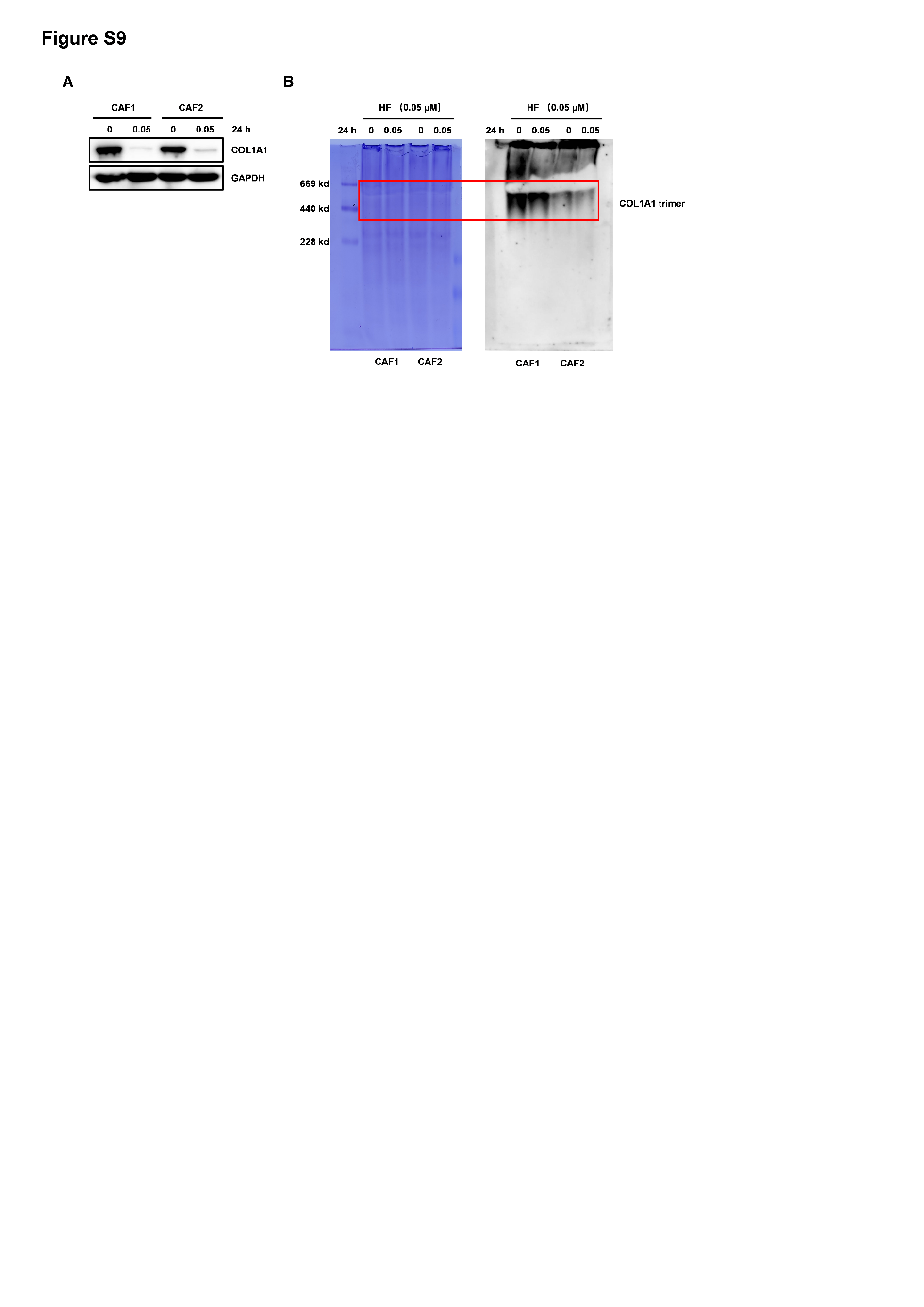


**Figure S9.** Halofuginone inhibits COL1A1 trimerization. A-B) HF treated CAF1 and CAF2, respectively as indicated, after 24 hours, the expression and trimerization of COL1A1 was detected by non-denaturing gel electrophoresis and Western blot.


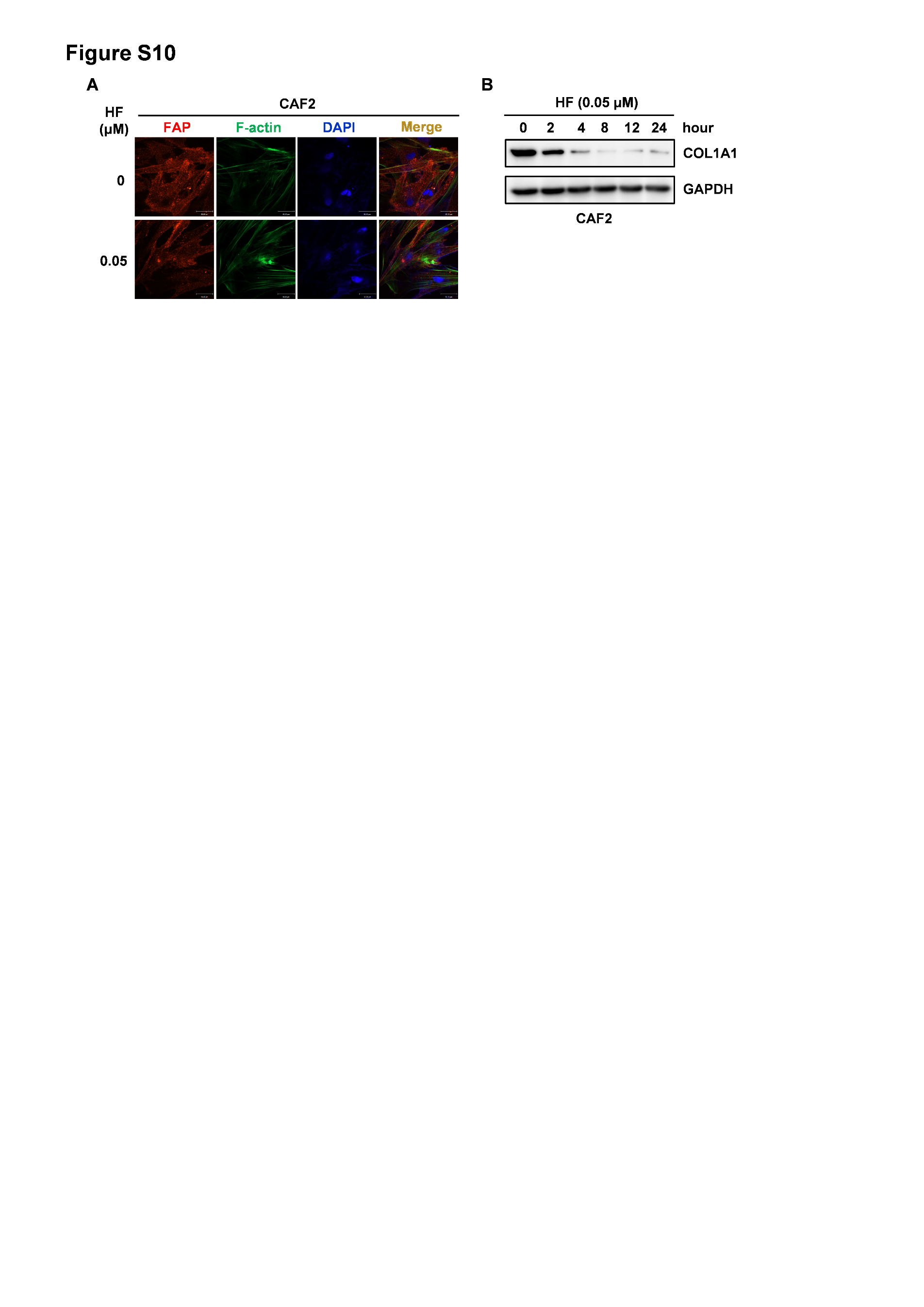


**Figure S10.** Halofuginone inhibits CAF2 markers expression. A) CAF2 was treated with 0.05 μ_M_ HF, 12 h after treatment, FAP (red), phalloidin-stained cytoskeleton (green), and DAPI-stained nuclei (blue) were detected by fluorescence microscopy. Scale bar, 30 µm. B) COL1A1 expression in CAF2 treated with 0.05 μ_M_ HF as indicated was detected by Western blot.


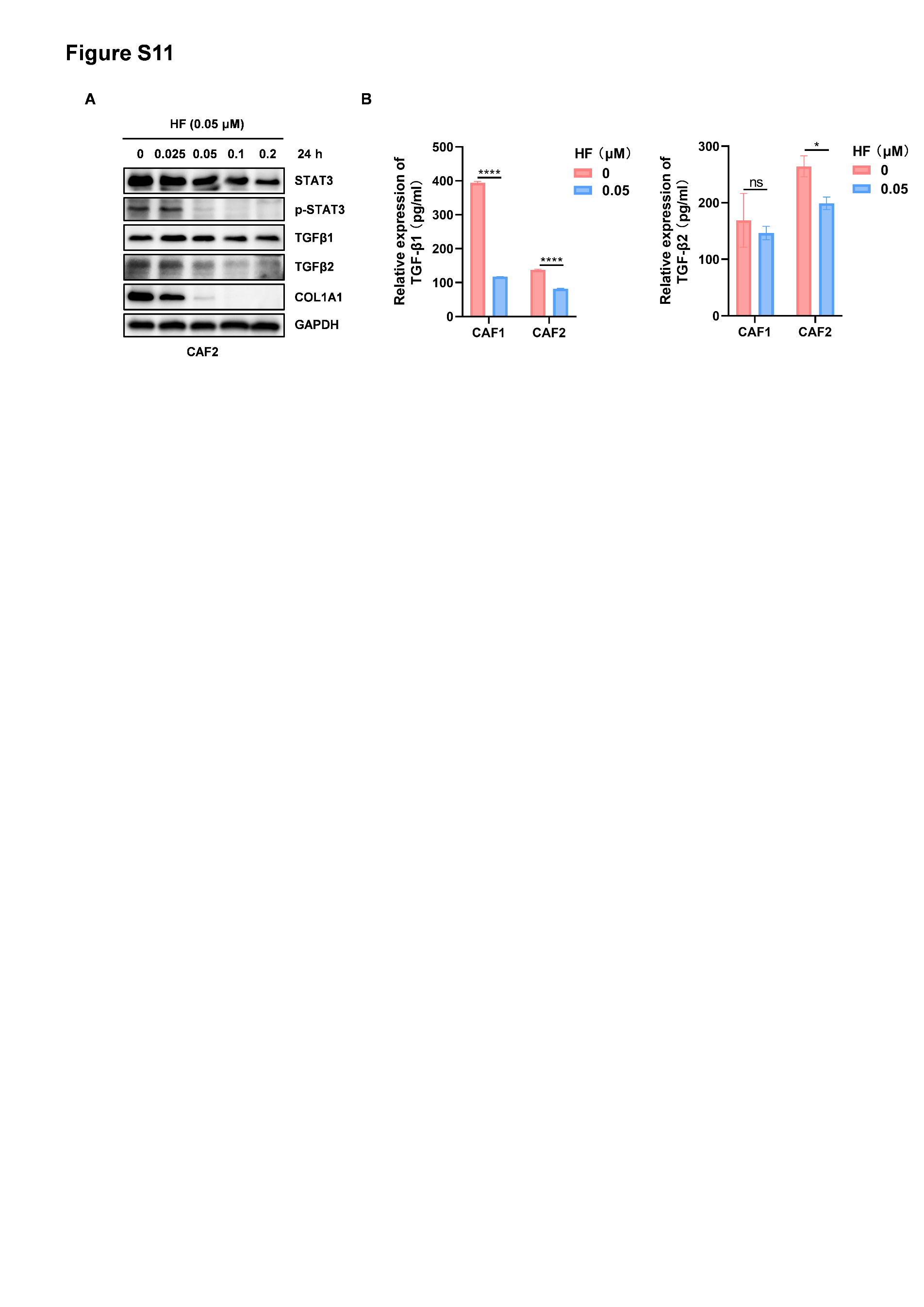


**Figure S11.** HF inhibited the expression of COL1A1 through STAT3-TGFβ axis in ovarian cancer CAFs. A) CAF1 were treated with 0.05 μ_M_ HF as indicated time. Expression of COL1A1 and p-STAT3 were detected by Western blot. B) Detected the activity of TGFβ1 and TGFβ2 in CAF1 and CAF2 conditioned media after 0.05 μ_M_ HF treatment for 24 h by ELISA.


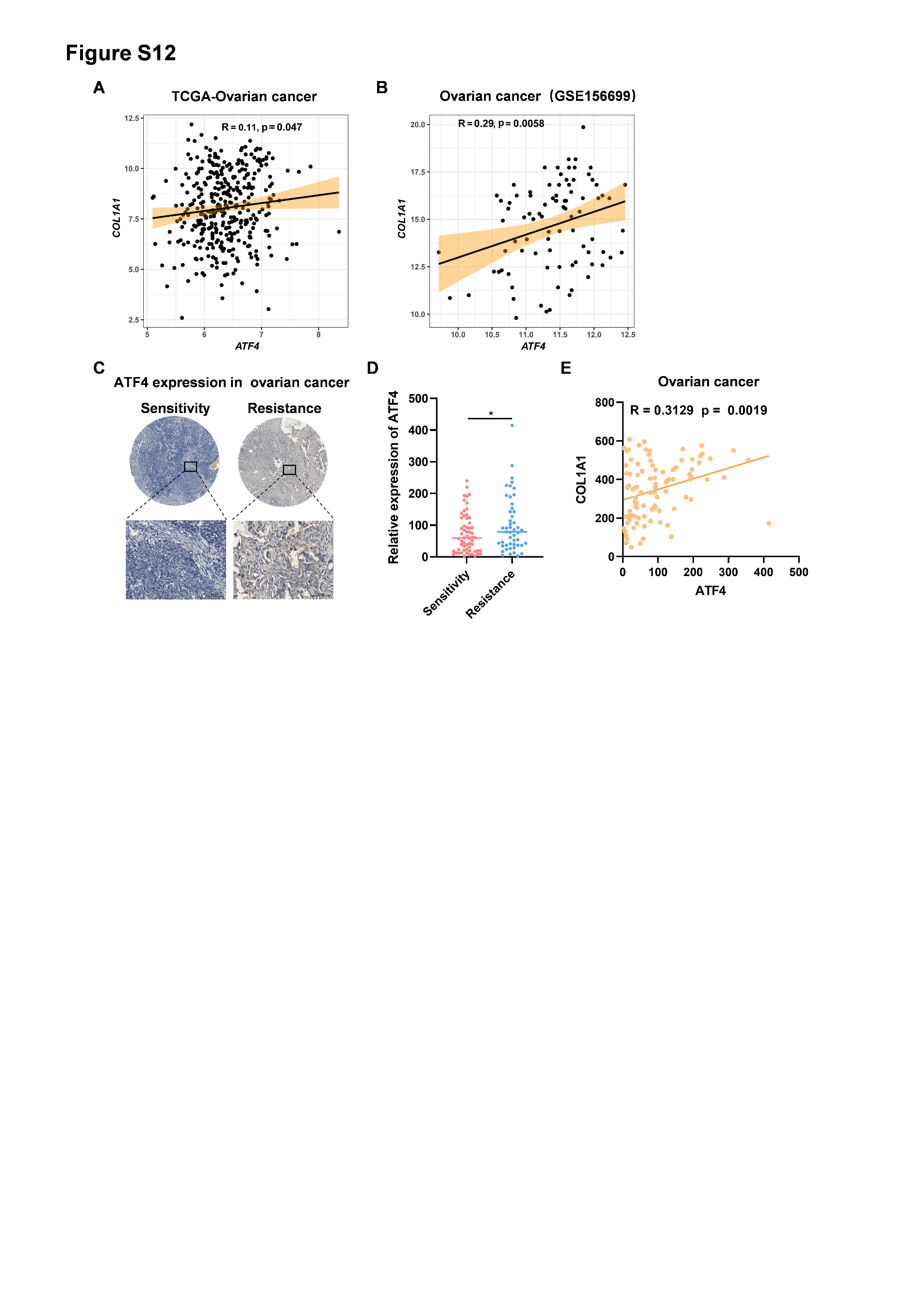


**Figure S12.** ATF4 regulated COL1A1 expression in ovarian cancer. A-B) The correlation between *ATF4* and *COL1A1* expression in ovarian cancer TCGA (A) and GSE156699 (B) datasets. C-D) ATF4 expression in ovarian cancer was detected by IHC using tissue microarray. Representative images of ATF4 expression (C, Scale bar, 50 μm). Analysis of ATF4 expression between in sensitivity and resistance groups (D). E) The correlation between ATF4 and COL1A1 expression in ovarian cancer.

**
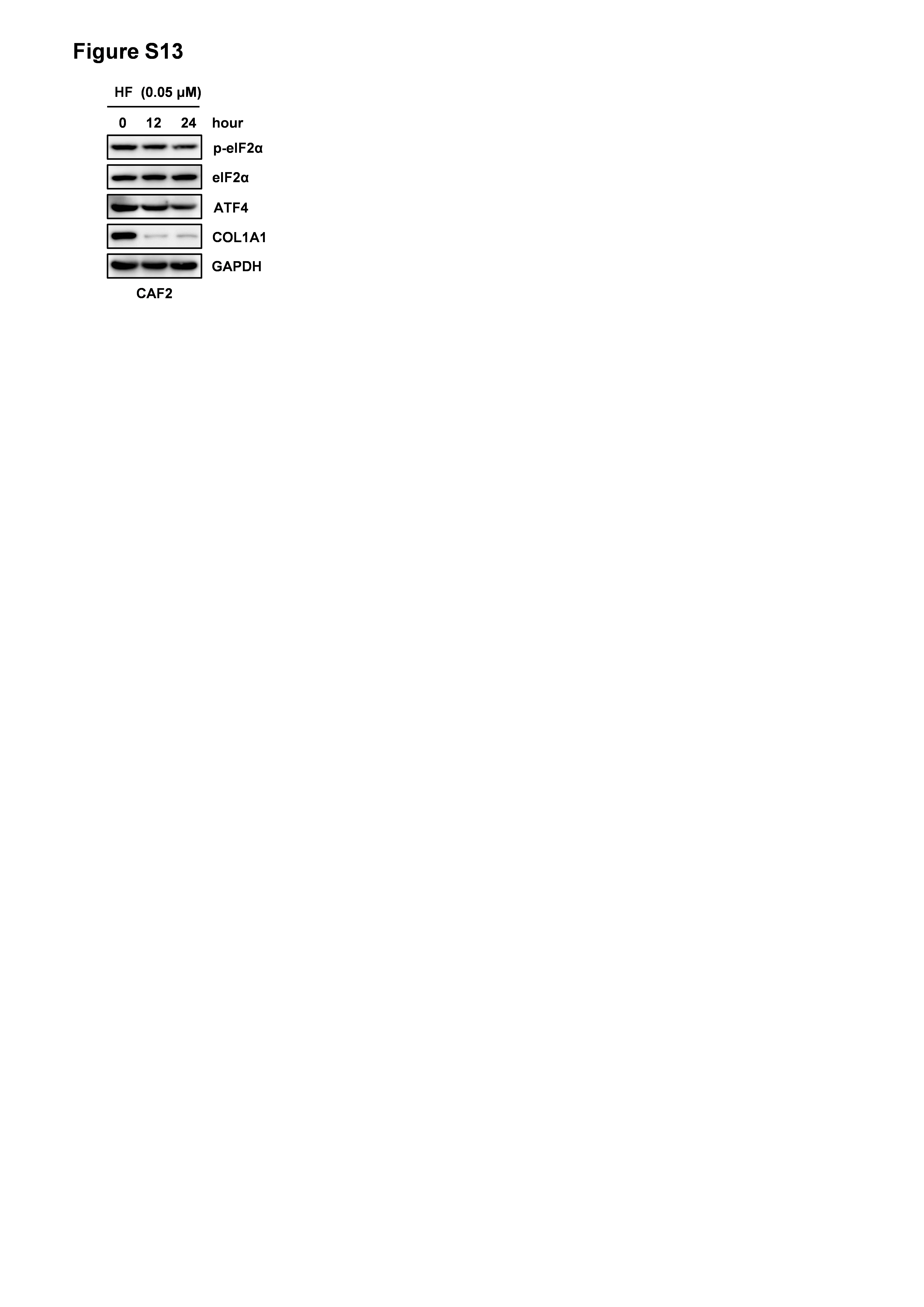
**

**Figure S13.** Halofuginone inhibits COL1A1 expression through eIF2α-ATF4 axis. CAF2 was treated with 0.05 μ_M_ HF as indicated. After treatment, expression of p-eIF2α, eIF2α, ATF4, and COL1A1 was detected by Western blot.

**
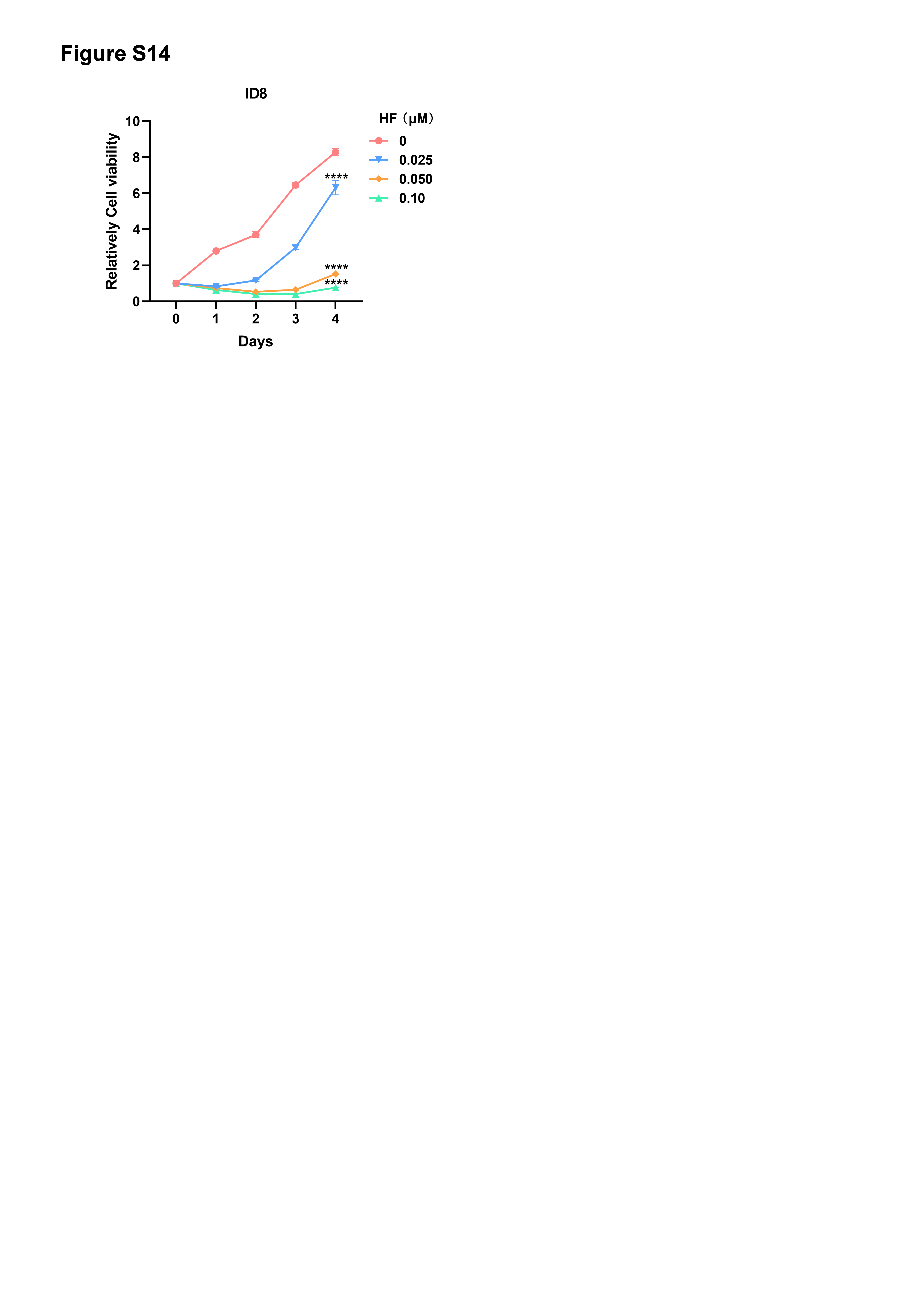
**

**Figure S14.** Halofuginone inhibits proliferation of ovarian cancer cell. Mouse ovarian cancer cells ID8 was treated with different concentration of HF as indicated, 24 h after treatment, cell viability was detected by CCK8 assay. Data was presented as mean ± SD (n = 3). Statistical analysis was performed by two-tailed, unpaired Student’s t-test, and two-way ANOVA with Tukey’s test, ****p < 0.0001.


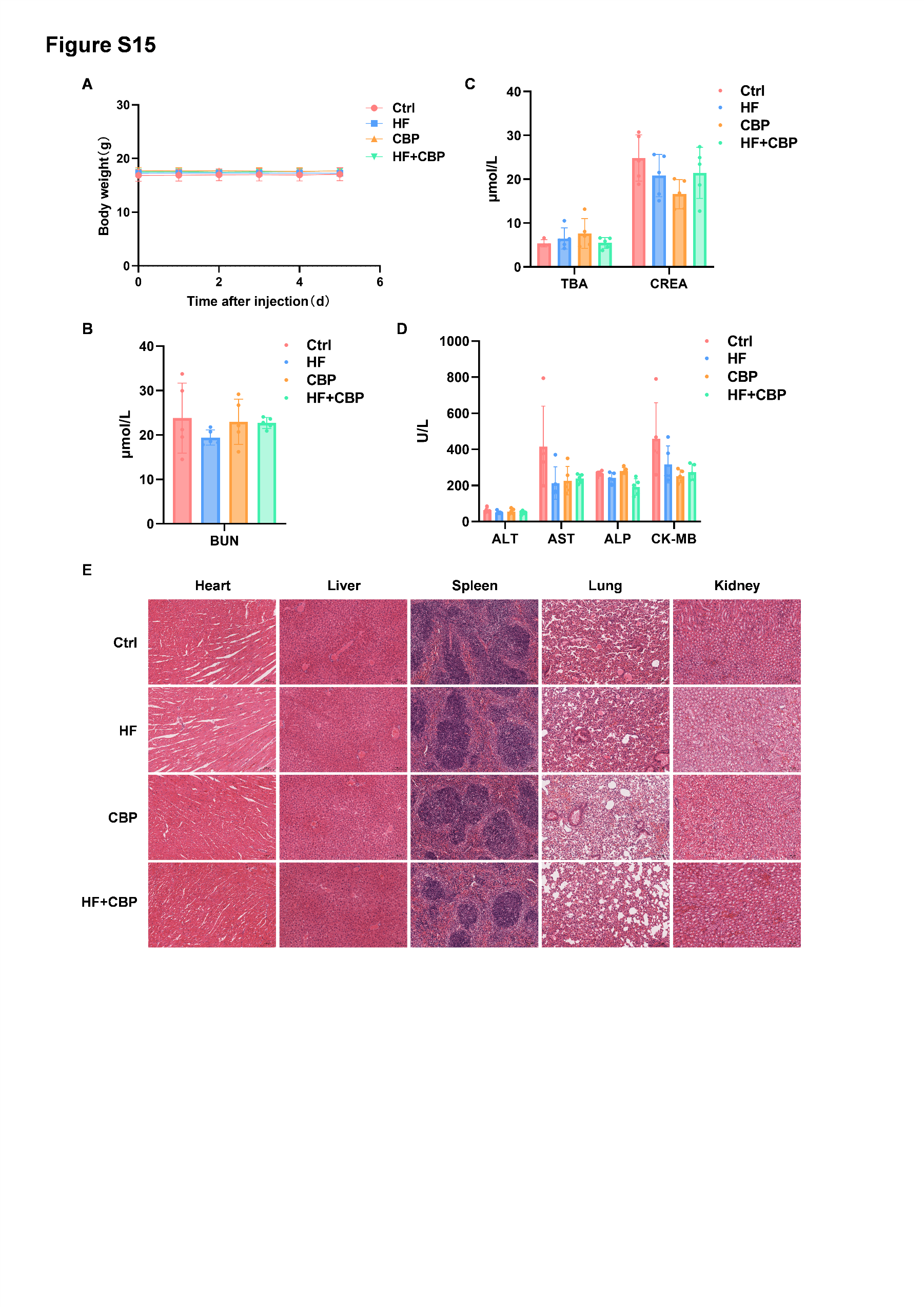


**Figure S15.** Safety evaluation of Halofuginone. A) Mice were weighed after treatment as indicated. B-D) Blood biochemical indexes reflecting heart, liver, and kidney functions were determined in the indicated groups. n = 5 mice per group. E) Hematoxylin and eosin (H&E) staining of major organs including heart, liver, spleen, and kidney. n = 3 mice per group, scare bar, 100 μm.

**
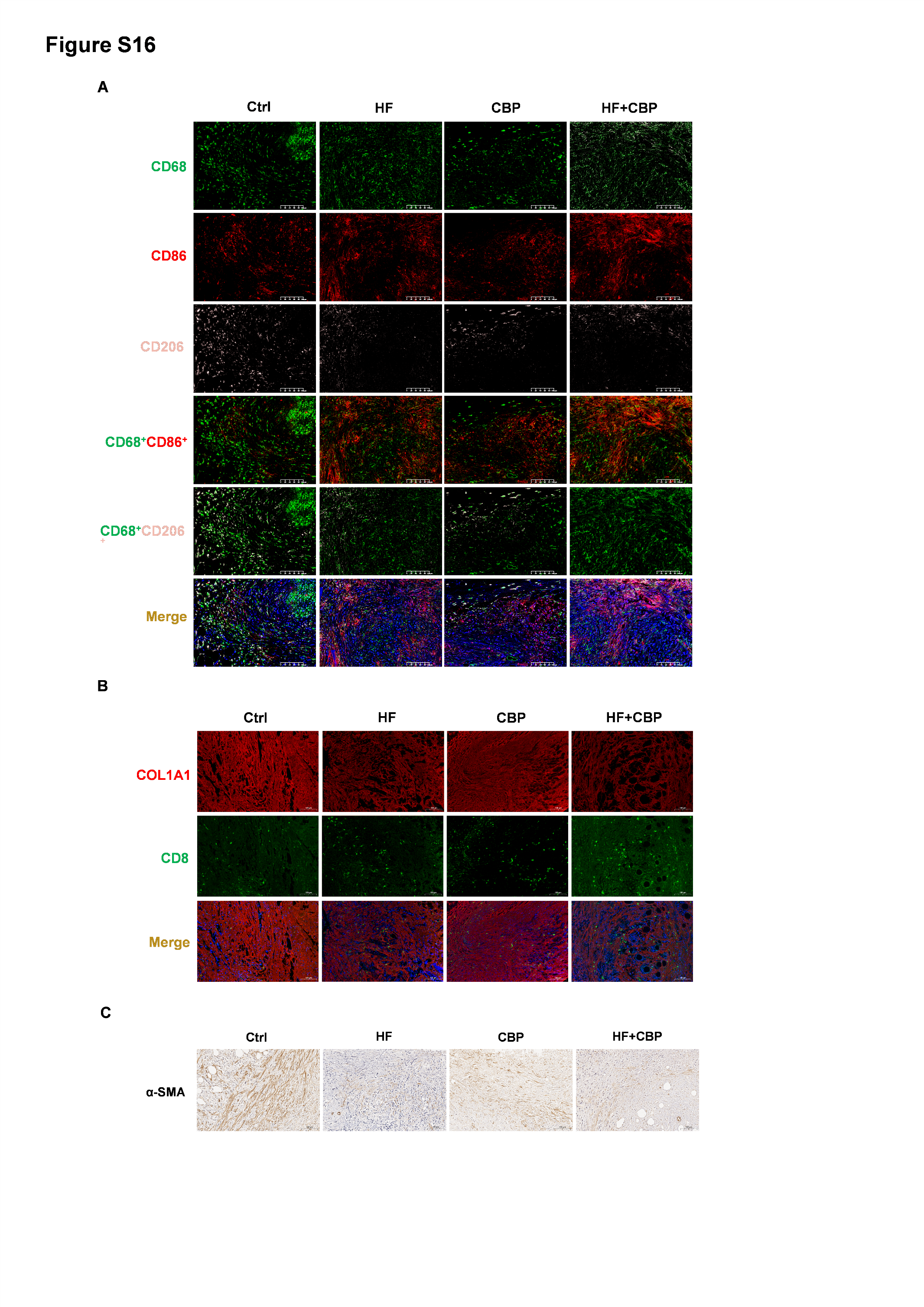
**

**Figure S16.** Halofuginone combined with Carboplatin reshapes TME. A) The expression of CD68 (green), CD86 (red), and CD206 (pink) in these tissues as indicated in Figure 6B was examined by mIHC. The representative images in each group as indicated. B) The expression of COL1A1 (red) and CD8 (green) was examined by immunofluorescence, and the representative images in each group as indicated, scale bar 100 µm (G). C) IHC assay of α-SMA expression in tumor-bearing ID8 cells as indicated in Figure 6A, scale bar 100 µm.


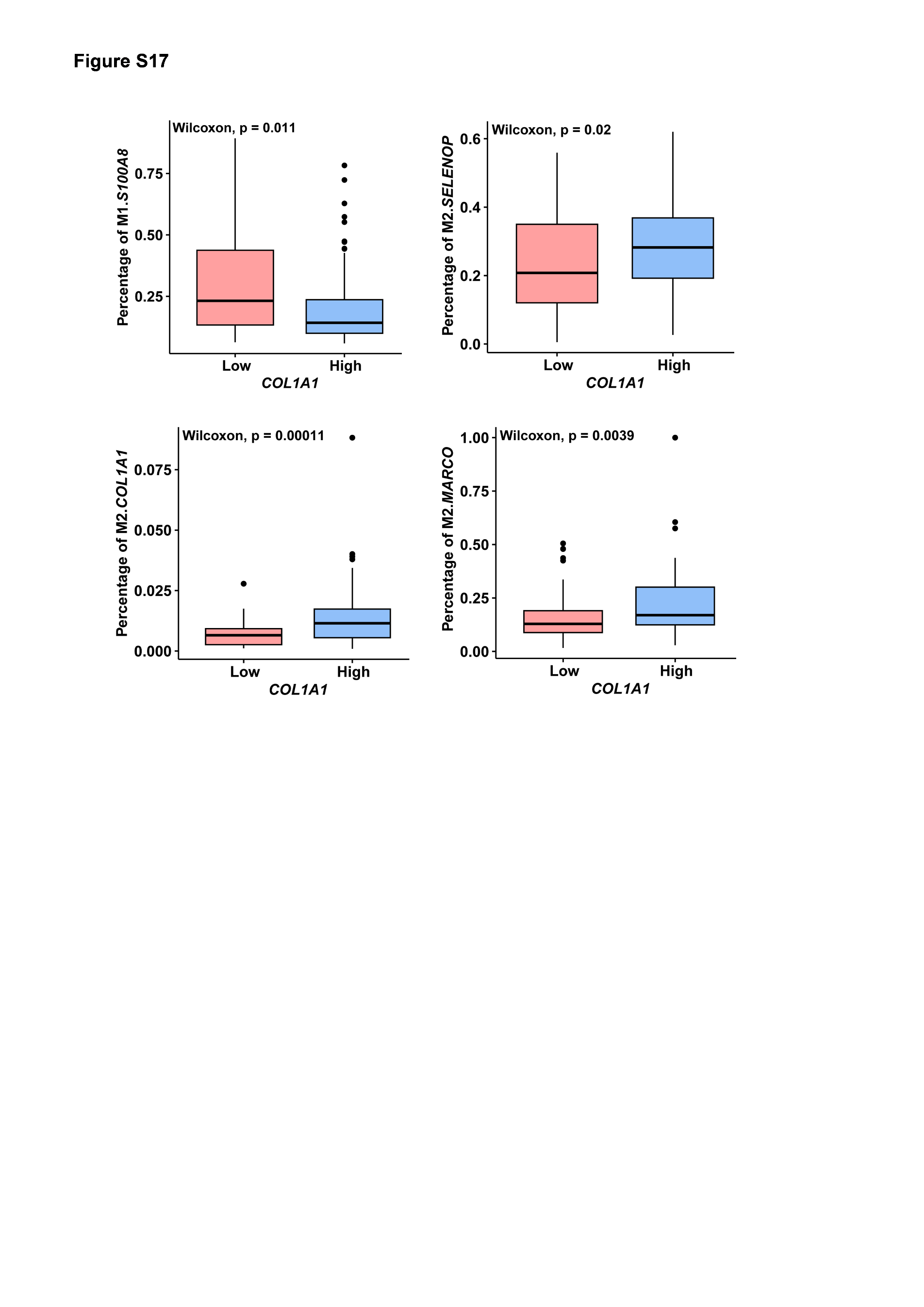


**Figure S17.** Correlation between tumor-associated macrophage type and COL1A1 in ovarian cancer. Analysis of the percentage of M1 and M2 macrophages in the COL1A1^High^ and COL1A1^Low^ groups.
